# Design of AI-Enhanced and Hardware-Supported Multimodal E-Skin for Environmental Object Recognition and Wireless Toxic Gas Alarm

**DOI:** 10.1007/s40820-024-01466-6

**Published:** 2024-07-29

**Authors:** Jianye Li, Hao Wang, Yibing Luo, Zijing Zhou, He Zhang, Huizhi Chen, Kai Tao, Chuan Liu, Lingxing Zeng, Fengwei Huo, Jin Wu

**Affiliations:** 1 State Key Laboratory of Optoelectronic Materials and Technologies and the Guangdong Province Key Laboratory of Display Material and Technology, School of Electronics and Information Technology, Sun Yat-Sen University, Guangzhou, 510275 People’s Republic of China; 2 Ministry of Education Key Laboratory of Micro and Nano Systems for Aerospace, School of Mechanical Engineering, Northwestern Polytechnical University, Xi’an, 710072 People’s Republic of China; 3 Research & Development Institute of Northwestern Polytechnical University in Shenzhen, Shenzhen, 518063 People’s Republic of China; 4 The Institute of Flexible Electronics (IFE, Future Technologies), Xiamen University, Xiamen, 361005 People’s Republic of China; 5 Key Laboratory of Flexible Electronics (KLOFE), School of Flexible Electronics (Future Technologies), Nanjing Tech University, 30 South Puzhu Road, Nanjing, 211816 People’s Republic of China; 6 State Key Laboratory of Transducer Technology, Shanghai, 200050 People’s Republic of China; 7 Guangdong Provincial Key Laboratory of Technique and Equipment for Macromolecular Advanced Manufacturing, Guangzhou, 510641 People’s Republic of China; 8 State Key Laboratory of Polymer Materials Engineering, Sichuan University, Chengdu, 610065 People’s Republic of China; 9 Guangdong Provincial Key Laboratory of Research and Development of Natural Drugs and School of Pharmacy, Guangdong Medical University, Dongguan, 523808 People’s Republic of China; 10 The First Dongguan Affiliated Hospital, Guangdong Medical University, Dongguan, 523808 People’s Republic of China; 11 Engineering Research Center of Polymer Green Recycling of Ministry of Education, College of Environment and Resources, Fujian Normal University, Fuzhou, 350007 People’s Republic of China

**Keywords:** Stretchable hydrogel sensors, Multimodal e-skin, Artificial intelligence, Post-earthquake rescue, Wireless toxic gas alarm

## Abstract

Post-earthquake rescue missions are full of challenges due to the unstable structure of ruins and successive aftershocks. Most of the current rescue robots lack the ability to interact with environments, leading to low rescue efficiency. The multimodal electronic skin (e-skin) proposed not only reproduces the pressure, temperature, and humidity sensing capabilities of natural skin but also develops sensing functions beyond it—perceiving object proximity and NO_2_ gas. Its multilayer stacked structure based on Ecoflex and organohydrogel endows the e-skin with mechanical properties similar to natural skin. Rescue robots integrated with multimodal e-skin and artificial intelligence (AI) algorithms show strong environmental perception capabilities and can accurately distinguish objects and identify human limbs through grasping, laying the foundation for automated post-earthquake rescue. Besides, the combination of e-skin and NO_2_ wireless alarm circuits allows robots to sense toxic gases in the environment in real time, thereby adopting appropriate measures to protect trapped people from the toxic environment. Multimodal e-skin powered by AI algorithms and hardware circuits exhibits powerful environmental perception and information processing capabilities, which, as an interface for interaction with the physical world, dramatically expands intelligent robots’ application scenarios.

**Supplementary Information:**

The online version contains supplementary material available at 10.1007/s40820-024-01466-6.

## Highlights

- A novel organohydrogel-based multimodal e-skin with excellent sensing performance for temperature, humidity, pressure, proximity, and NO_2_ is proposed for the first time, showing powerful sensing capabilities beyond natural skin.
- The developed multimodal e-skin exhibited extraordinary sensing performance at room temperature, including fast pressure response time (0.2 s), high temperature sensitivity (9.38% °C^-1^), a wide range of humidity response (22%–98% RH), high NO_2_ sensitivity (254% ppm^-1^), a low detection limit (11.1 ppb NO_2_) and the abilities to sense the proximity of objects accurately, which are yet achieved by previous e-skins.
- The multimodal e-skin was combined with the deep neural network algorithm and wireless alarm circuit to achieve zero-error classification of different objects and rapid response to NO_x_ leak incidents, proving the feasibility of the e-skin-assisted rescue robot for post-earthquake rescue.

**Supplementary Information:**

The online version contains supplementary material available at 10.1007/s40820-024-01466-6.

## Graphical abstract

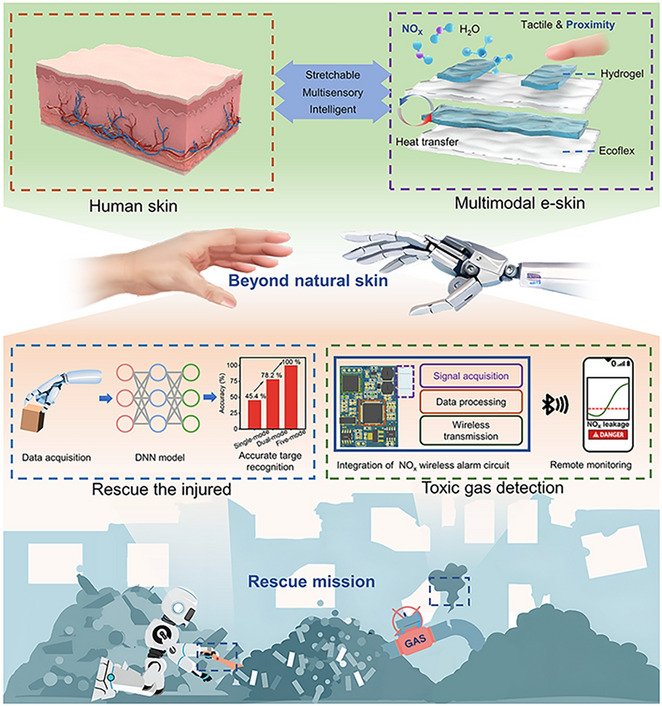

## Introduction

Earthquakes are one of the most severe natural disasters in the world. According to statistics [1], from 2000 to 2023, the earthquake caused around $816 million in economic losses and 2.43 million casualties worldwide. Losses caused by earthquakes not only originate from building damage and casualties during earthquakes but also come from the difficult post-earthquake rescue work, which consumes huge manpower, material, and financial resources [2, 3]. Miniature rescue robots can be remotely controlled to search, dig, and rescue buried persons in narrow and complex terrain, expected to replace rescuers in dangerous missions [4–6]. Most current rescue robots search for the location of buried people through infrared imaging and acoustic wave detection. However, during the rescue process, a rescue robot without tactile perception cannot distinguish interference objects such as debris from the trapped person through touching and grasping since most of the victims’ body is covered by obstacles, significantly affecting rescue efficiency. In addition, severe earthquake disasters may damage chemical companies’ production equipment and storage tanks, causing toxic substances to leak into the external environment and posing life threats to rescuers and buried people [7, 8]. Thus, endowing rescue robots with powerful environmental perception capabilities has enormous and profound positive significance for improving rescue efficiency and reducing rescue costs. Multimodal sensors, which combine multiple sensing functions into a single device, are considered to be a potential means to endow rescue robots with powerful sensing capabilities. However, integrating multimodal sensors onto the irregular rigid shell of the robot is still a challenge that needs to be solved.

The skin is the largest organ of the human body and a powerful natural multisensory system that can sense various environmental stimuli such as pressure, temperature, and humidity [9, 10]. Recently, the research on flexible electronics has made significant progress. Relative technologies are widely applied in electromagnetic shielding, health detection, and robot perception [11–14]. Inspired by human skin, researchers are committed to using emerging technologies based on flexible electronic materials and bionic electronic devices to develop artificial skin with flexibility, elasticity and multi-sensing functions comparable to natural skin. By attaching the e-skin to the rigid shell of the robot, it is expected to reproduce the touch, temperature, moisture, and other sensations of natural skin on the rescue robot, realizing accurate identification of buried people in non-visual environments by touching and grasping objects. In addition, the extreme plasticity of e-skin makes it possible to surpass natural skin in all aspects. Integrating more sensing modules on the e-skin or improving the performance of each module can gain far better sensing properties than natural skin. For example, the integration of gas sensing modules can enhance environmental awareness of the e-skin, thereby timely detecting potential dangers in the environment, such as the leakage of toxic gases.

Due to the excellent potential and application prospects of e-skin, much research on e-skin has been carried out to reproduce the sensory function of real skin and even obtain performance beyond it [15, 16]. Among all the skin functions, the tactile sense is one of the essential ones. A small electric signal is generated when the skin’s tactile receptors are mechanically stimulated. Then, the electric signal is transmitted to the nerve center through nerve fibers, generating tactile sense [17]. The general idea of realizing the tactile sensation is to sense mechanical stimuli by integrating flexible sensors on the e-skin to convert these stimuli into tactile signals [18–20]. For example, Boutry et al. of Bao’s group developed a capacitive bionic soft e-skin, inspired by the structure of human skin [21]. The upper and lower plates of the capacitor are carbon nanotube–polyurethane (CNT-PU) composite electrodes with unique hill shapes and pyramid structures, respectively, which are used to mimic the interlocked dermis-epidermis in human skin. The proposed e-skin can measure and distinguish normal and tangential forces well through real-time capacitance changes of the spatial capacitor array. Zhu et al. tried constructing a skin–electrode interface by attaching sensing electrodes (SE) of Au-deposited micropillar polydimethylsiloxane (PDMS) and counter electrodes (CE) of Au film to human skin [22]. The skin–electrode interface shows different capacitance values under different pressures, exhibiting high resolution and capable of feeling touch and detecting fingertip pulse. Although using human skin instead of the traditional dielectric layer can reduce device assembly complexity, changes in skin temperature and humidity, and even the relative distance between SE and CE, can cause performance fluctuations.

Although reported e-skin can well achieve pressure sensing functions, it is still far from the perfect reproduction of natural skin from all aspects. With multisensory capabilities, human skin is not only sensitive to mechanical stimuli but also responsive to ambient temperature and humidity. The rises or falls of ambient temperature/humidity can be quickly felt through the skin. A dry and low-temperature environment will accelerate the loss of skin moisture, making the skin feel dry and tight. In contrast, a humid and high-temperature environment will lead to the vigorous secretion of sweat glands, resulting in greasy skin. Multimodal sensors that mimic skin functions have been extensively researched and plenty of advances have been made [23–25]. Sensing materials with multi-stimulus response characteristics is the key to building multimodal sensors beyond natural skin [26]. Zhang et al. proposed a PANSion material through a strategy of silver-nitrile complexes with in situ-grown AgNPs, successfully realizing the dual sensing function of mechanical and thermal stimuli [27, 28]. In order to realize the multi-sensing function of e-skin, An et al. exploited silver nanofiber-silver nanowire hybrid networks as high-performance transparent electrodes and a high-κ and transparent cellulose nanofiber (CNF) film as the dielectric layer to construct a capacitive pressure sensing array for fingerprint detection [29]. Meanwhile, the capacitor array was integrated with a pressure-sensitive field-effect transistor (FET) and a poly(3,4-ethylenedioxythiophene): polystyrene sulfonate-based temperature sensor, thereby achieving multifunctional detection of finger pressure and skin temperature. Yang et al. successfully constructed a pressure–temperature robot skin by sandwiching the iontronic film between flexible electrodes [30]. Multimodal sensing and signal decoupling are achieved using rationally designed electrodes and a resistance–capacitance dual-measurement strategy. Zou et al. incorporated conductive silver nanoparticles (AgNPs) into polyimide to improve its conductivity and fabricated an integrated tactile, flow, temperature, and humidity platform based on the conductive polyimide film [31]. Notably, this e-skin reproduces the repairability of natural skin, and the repaired e-skin exhibits mechanical and electrical properties comparable to the original device.

Previous research mainly focused on reproducing the structure or function of natural skin, research on sensory functions beyond natural skin is rarely reported. Hydrogel materials have regulable mechanical properties and can gain multi-stimuli responsiveness through structural design and component adjustment of hydrogel engineering [32–34], showing great application prospects in wearable sensing, human–machine interaction, and robot perception [35–41]. The organohydrogel-based multimodal e-skin we proposed in this work not only restores natural skin’s function in sensing mechanical stimulation, temperature, and humidity but also possesses characteristics beyond natural skin-precise perception of object proximity and NO_2_ gas. The introduction of glycerol solvent provides PVA-CNF organohydrogel with excellent anti-freezing and anti-drying ability, successfully realizing wide range temperature detection while avoiding water freezing or evaporation. The PVA-CNF polymer network contains a large amount of O–H inside, which can physically bond with free water molecules through hydrogen bonds, resulting in excellent humidity sensitivity. PVA-CNF organohydrogel with good mechanical properties and certain conductivity can be exploited as the plate electrode of the pressure and proximity module. Rich water environment and porous polymer network inside organohydrogel provide good channels for ion transport in the NO_2_ reaction. Although PVA-CNF organohydrogel exhibits lower electrical conductivity compared with some ionic conductive hydrogel, its advantages in anti-freezing and moisture retention, transparency, mechanical properties, multisensory and self-calibration make it stand out among hydrogel-based e-skins (Table S1) [42–46]. Traditional environmental humidity or temperature calibration relies on complex sensor arrays and algorithms. For example, Li et al. proposed a hygroscopic materials-based wearable sensing array and introduced backpropagation neural network (BP-NN) and partial least squares (PLS) algorithms to achieve accurate prediction of gas concentration over a wide range of relative humidity [47]. In our work, by monitoring the temperature or humidity changes in the environment through temperature and humidity modules, the multimodal e-skin can calibrate other sensing modules without needing external devices or algorithms, ensuring normal operation under different temperature and humidity conditions. The e-skin based on sensitive material of polyvinyl alcohol-cellulose nanofiber (PVA-CNF) organohydrogel exhibits fast pressure response time (0.2 s), high temperature sensitivity (9.38% °C^−1^), a wide range of humidity response (22%–98% RH), low gas detection limit (11.1 ppb NO_2_), high NO_2_ sensitivity (254% ppm^−1^) and the ability to sense the proximity of objects accurately. Furthermore, the e-skin based on stretchable PVA-CNF organohydrogel and Ecoflex has mechanical properties similar to natural skin and can well adapt to arbitrary complex surfaces, including the rigid shell of a robot, endowing the robot with perceptions comparable to or even better than human skin.

After being installed on the fingers of commercial pneumatic hands, AI-enhanced multimodal e-skin successfully recognizes five different objects with 100% accuracy through grasping. Besides, the multimodal e-skin was combined with a well-designed wireless gas alarm circuit to achieve real-time monitoring of the ambient NO_2_ concentration and rapid detection of NO_x_ leak events. The rescue robot, integrated with the AI-enhanced multimodal e-skin and NO_x_ wireless monitoring system, can effectively search and rescue the trapped person in non-visual environments while ensuring their safety. The combination of multimodal e-skin, AI algorithms and dedicated hardware circuits has dramatically expanded the robot’s sensing capabilities and improved its performance in complex tasks, providing a new direction for the advancement of future intelligent robotics.

## Experimental Section

### Synthesis of PVA-CNF Organohydrogels

First, deionized water and glycerol were mixed at a mass ratio of 1:1 to obtain an H_2_O-Gly mixed solution. CNFs and PVA were successively added to the H_2_O-Gly mixed solution, and the mass ratio of CNFs, PVA and H_2_O-Gly mixture was 1:15:100. The resulting mixture was then stirred in a sealed environment at 100 °C for 5 h for homogeneous mixing. Finally, the precursor solution was spin-coated on an aluminum substrate at 2000 rpm for 60 s and kept at room temperature for 12 h to obtain a PVA-CNF organohydrogel film.

### Fabrication of Multimodal e-Skin

Ecoflex precursors (A and B) were mixed at a mass ratio of 1:1, spin-coated on an aluminum substrate at 500 rpm for 60 s and kept at room temperature for 30 min to cure. After being cut into suitable sizes and affixed with conductive tapes on both ends as electrodes, the organohydrogel film was placed on the Ecoflex substrate as a temperature sensing module. Another Ecoflex layer was spin-coated at 500 rpm for 60 s and wrapped around the hydrogel film to avoid interference from ambient humidity. Afterward, two organohydrogel films connected to silver wires at both ends were placed on the uncured Ecoflex as humidity sensing or NO_2_ sensing modules and the top plate electrode of the pressure/proximity module, respectively. When the top Ecoflex layer was cured, the organohydrogel films above could be well fixed. In addition, the top organohydrogel film and the Ecoflex-wrapped organohydrogel film are perpendicular to each other, forming a hydrogel-Ecoflex-hydrogel sandwich structure in the overlapping area for pressure and proximity sensing (Fig. S1).

### Sensing Performance Characterization

#### Humidity and Temperature Sensing Measurement

Different types of saturated salt solutions were put into closed containers. The relative humidity above the liquid level depends on the type of saturated solution, thereby obtaining a series of environments with different relative humidity. Additionally, the containers were placed in a constant temperature drying oven. By changing the temperature of the drying oven, the response of the humidity module at different temperatures is measured. The relative humidity of the surrounding environment of different salt solutions at various temperatures is shown in Table S2. An AC voltage with an amplitude of 1 V and a frequency of 200 Hz was applied to two electrodes of the humidity module, and an LCR meter (2832; Tonghui Electronic Co., Ltd.) was used to record the resistance change of the module. For temperature sensing characterization, the e-skin was placed above a hot plate. An AC voltage with an amplitude of 1 V and a frequency of 200 Hz was applied to the electrodes at both ends of the temperature module, and an LCR meter was used to record its resistance changes at different temperatures.

#### Pressure and Proximity Sensing Measurement

Pressure sensing tests were performed on a homemade motorized mobile platform (Zolix, SC300-1B), and a force meter (HANDPI Co. Ltd, HP) was used to record the force exerted on the module. This self-made electric mobile platform can also control the distance between the object and the proximity module when conducting the proximity sensing test. The capacitance value was measured through an LCR meter by applying an AC voltage with an amplitude of 1 V and a frequency of 200 kHz to the electrodes at both ends of the test module. The device is placed on a temperature-adjustable hot plate, by repeating the pressure and proximity test process described above, the performance of the pressure and proximity module at different temperatures is obtained.

#### Gas Sensing Test

A DC bias voltage of 0.5 V was applied to the electrodes at both ends of the NO_2_ module. The reaction current signal was recorded by an analyzer (Keithley 2400). The target gas (10 ppm NO_2_) was mixed with pure N_2_ through a digital mass flow controller to obtain different NO_2_ concentrations. Mixed test gases with different NO_2_ concentrations were injected into the chamber while the current signal of the module was recorded, and then the chamber was cleaned with N_2_ for signal recovery. In order to control the humidity during the test, wet gas (acquired by injecting dry gas into the water to bubble) and dry gas were mixed in different proportions to obtain a series of mixed gases with different relative humidity. The relative humidity of dry and wet gas mixtures with different flow rate ratios was calibrated with a hygrometer (Table S3). The closed chamber was placed in dimethyl silicone oil for oil bath heating to adjust the temperature, and the module’s response to NO_2_ at different temperatures was measured.

### Finite Element Analysis

The object detection process of the proximity module was simulated through COMSOL software. The constructed 3D model is identical to the actual structure of the e-skin. The size of the organohydrogel film wrapped by Ecoflex was 20 × 4 mm^2^, and the other two organohydrogel films were placed on the top of the device, with a size of 8 × 3 mm^2^. The thickness of hydrogel films was 62.47$\upmu\text{m}$. The dimensions of the Ecoflex encapsulation layer were 30 × 20 × 2.5 mm^3^. At 25 °C and 60% RH, the conductivity of the original organohydrogel was 0.07828 S m^−1^, and its relative permittivity was measured to be 34.8 through the plate capacitance method. Insulating elastomer Ecoflex has an electrical conductivity of approximately 0 and a relative permittivity of 2.65. A conductive cylinder with a radius of 1.7 mm and a height of 10 mm was modeled to simulate the measured object and was set to 0 V as the ground terminal. An AC voltage with an amplitude of 1 V and a frequency of 200 kHz was applied between the upper and lower gel plates. The external space of the e-skin was an air domain with a conductance of 0 and a relative permittivity of 1. The finite element method was used to simulate the electric field distribution and potential distribution in the surrounding space, as well as the capacitance of the proximity module.

### AI Algorithms for Object Recognition

#### Data Acquisition and Processing

Commercial pneumatic gloves, with multimodal e-skin installed at the end of each finger, simulated robot hands for object grasping. The strength and speed of each grip were set to be constant. The robot hand grabbed five objects, including plastic bottles, rubber prosthetic hands, heated rubber prosthetic hands, dry wood, and wet wood. Each kind of object was grabbed for 20 times. The e-skin’s temperature, humidity, and pressure/proximity responses for each channel were read using the above data measurement methods. The temperature, humidity, and pressure/proximity response signals collected on the five fingers were extracted, and a dataset with 15 data features and 100 data samples was obtained. Shuffling the order of the dataset made the training results of the model more generalizable. After completing the data cleaning, the data were divided into a training set and a test set with a ratio of 1:1. In order to make model training converge faster, Z-score normalization is performed on the features of the data.

#### DNN Model Construction and Training

We implemented the deep neural network for the robot hand to conduct classification on Python 3.10.11 using the TensorFlow framework. The DNN model consists of an input layer, two fully connected hidden layers, and an output layer. The number of neurons in the input and hidden layers is 15 and 50, respectively, and their activation function is ReLu. The number of neurons in the output layer is 5, and its activation function is Softmax. In order to optimize the DNN model, we used the adaptive Adam optimization algorithm for model training, setting the initial learning rate to 0.001. The network was trained for 200 epochs using the training set. Finally, the test set was input into the trained model to evaluate its recognition accuracy for grasped objects.

## Result and Discussion

### Design of AI-Enhanced and Hardware-Supported Multimodal e-Skin

**Fig. 1:**
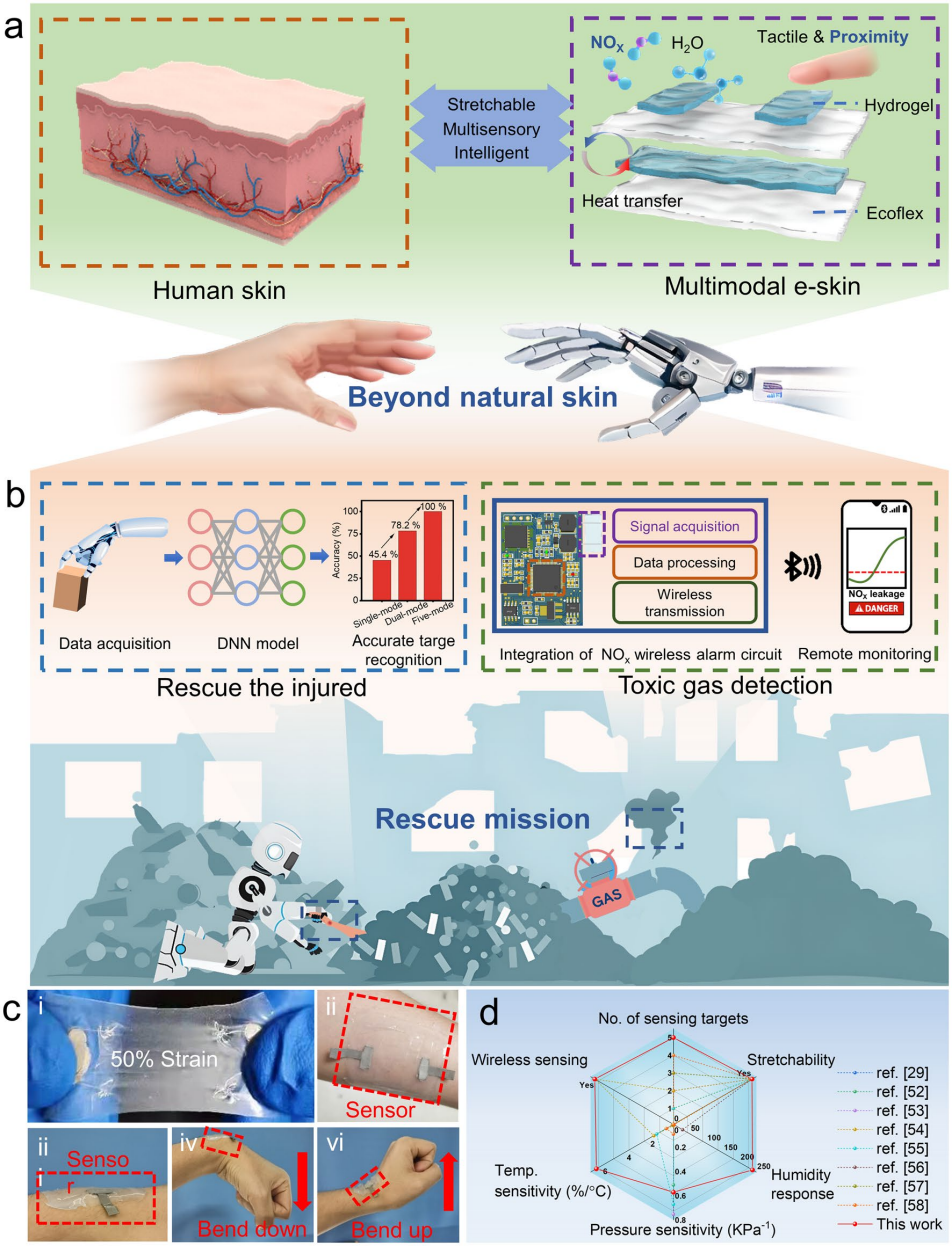
Schematic illustration and photographs for the properties and applications of multimodal e-skin. **a** E-skin based on PVA-CNF organohydrogel can not only sense traditional temperature, humidity, and pressure information but also perceive the proximity of objects and NO_2_ in the environment, beyond the capacity of natural skin. **b** Combination of multimodal e-skin and the DNN model enables rescue robots to accurately identify buried people at earthquake disaster sites. Furthermore, the integrated NO_x_ monitoring system, comprised of the multimodal e-skin and the gas alarm circuit, endows the intelligent robot with the function of monitoring toxic gases. **c** (i) Photograph of 50% tensile strain of multimodal e-skin. (ii) Photograph of the transparent multimodal e-skin attached to human skin. (iii–vi) Photographs show the e-skin being attached to a joint and following its movement without detaching. **d** Capability radar comparing the performance of the state-of-the-art e-skin

Human skin is a highly sophisticated intelligent sensing system that can accurately sense external temperature, pressure, humidity and other information and transmit it to the nervous system. The designed PVA-CNF organohydrogel-based e-skin can not only sense traditional temperature, humidity, and pressure information but also has the ability to detect the proximity of objects and environmentally dangerous gases, which significantly expands the application scenarios of e-skin (Fig. 1a).

The multimodal e-skin consists of stacked layers of different materials. The whole fabrication process of multimodal e-skin is shown in Fig. S1a and the Methods section. Figure S1b shows the relative positions of five different sensing modules. Wherein, conductive tapes and organohydrogel film serve as electrodes and the sensitive layer of conductive temperature sensor, respectively. The temperature sensing module is wrapped in Ecoflex film to isolate it from other environmental factors. The conductive temperature module detects environmental temperature by recording the conductance changes of the organohydrogel. Two organohydrogel films above the temperature module are connected to silver wires and exposed to the air, and are driven by the alternating current (AC) or direct current (DC) voltage sources to sense the humidity or NO_2_, respectively. Different driving signals are exploited to preclude the cross-talking. In humidity sensing, the response is based on conductance variation. In NO_2_ sensing, the current signal is derived from the Faradaic current generated by the electrochemical reaction of gas molecules at the electrode-gel interface, which reflects the NO_2_ level in the air. An Ecoflex film is sandwiched by another top organohydrogel film and inner organohydrogel films that are vertically overlapped to serve as capacitive pressure and proximity sensors. When the module is subjected to pressure, geometric deformation occurs, leading to a change in capacitance. In proximity sensing mode, the changing distance between the object and module will cause the electric field redistribution, ultimately resulting in capacitance change. The multimodal e-skin not only reproduces the soft and stretchable mechanical properties of human skin but also expands more sensing types, such as the toxic gas and proximity. This organohydrogel-based e-skin has sensory functions far beyond natural skin and is expected to be used in more diverse application scenarios.

Robots integrated with multimodal e-skin possess powerful multisensory capabilities and can perform various complex tasks by collecting abundant external information (Fig. 1b). For example, at earthquake disaster sites, rescue robots need to search for the victims in complex collapsed terrain and carry out rescue operations. A robot hand equipped with multimodal e-skins at the end of its fingers can collect information by grasping different objects. The collected temperature, humidity, pressure and proximity response signals are input into the trained DNN model to achieve accurate object classification. Compared with single-modal or dual-modal perception, multimodal perception can significantly improve the robot’s object recognition rate from 45.4% to 100%. The intelligent sensing system constructed by combining multimodal e-skin and DNN model enables the rescue robot to accurately identify the buried persons at the earthquake site and carry out further rescue. It is worth noting that earthquakes may damage the production equipment and storage tanks of chemical factories, causing leakage of toxic chemicals, especially harmful gases, which often bring danger to rescuers wherein nitrogen oxides are typical toxic gases in industrial production [48]. Even exposure to extremely low concentrations of NO_2_ can cause chronic damage to the human body’s central nervous and respiratory systems [49, 50]. The integrated NO_x_ monitoring system, which combines the multimodal e-skin and the gas alarm circuit, endows the rescue robot with the function of sensing toxic gases. Users can employ a self-programming App to communicate with the rescue robot through wireless Bluetooth technology, thereby obtaining the real-time NO_2_ concentration at the rescue site and ensuring the safety of rescuers and the buried victims.

The multimodal e-skin we proposed not only perfectly reproduces the multisensory functions of natural skin but also has flexibility and stretchability comparable to it. The multimodal e-skin can be stretched to 50% of its original length without any damage, demonstrating its excellent mechanical properties (Fig. 1c(i)). As can be seen from Fig. S2, Young’s modulus of multimodal e-skin is 84.93–218.30 kPa, which is the same order of magnitude as Young’s modulus of human skin (26.5 kPa at 50% strain) [51]. The breaking strain of the e-skin is 264%, reflecting the high stretchability. As can be seen from Fig. S3, with great transparency, the multimodal e-skin can be attached to a university logo pattern without affecting its appearance. Moreover, the soft e-skin shows excellent conformity to human skin surfaces and adapts well to the bending strain of the joint. (Fig. 1c(ii–vi)). Through device structural design and multiple sensing mechanisms, the multimodal e-skin proposed in this work exhibits significant advantages in sensing type, wireless monitoring, environmental object recognition, stretchability, and sensing performance compared with other reported e-skins (Fig. 1d, Table S4) [29, 52–58]. The pressure, humidity and temperature sensing functions, which are essential in natural skin, were selected as metrics for performance comparison. Multimodal e-skin, powered by AI algorithms and hardware circuits, exhibits properties far beyond natural skin and can significantly improve rescue efficiency in earthquake disasters while reducing rescue losses.

### Humidity and Temperature Modules

**Fig. 2:**
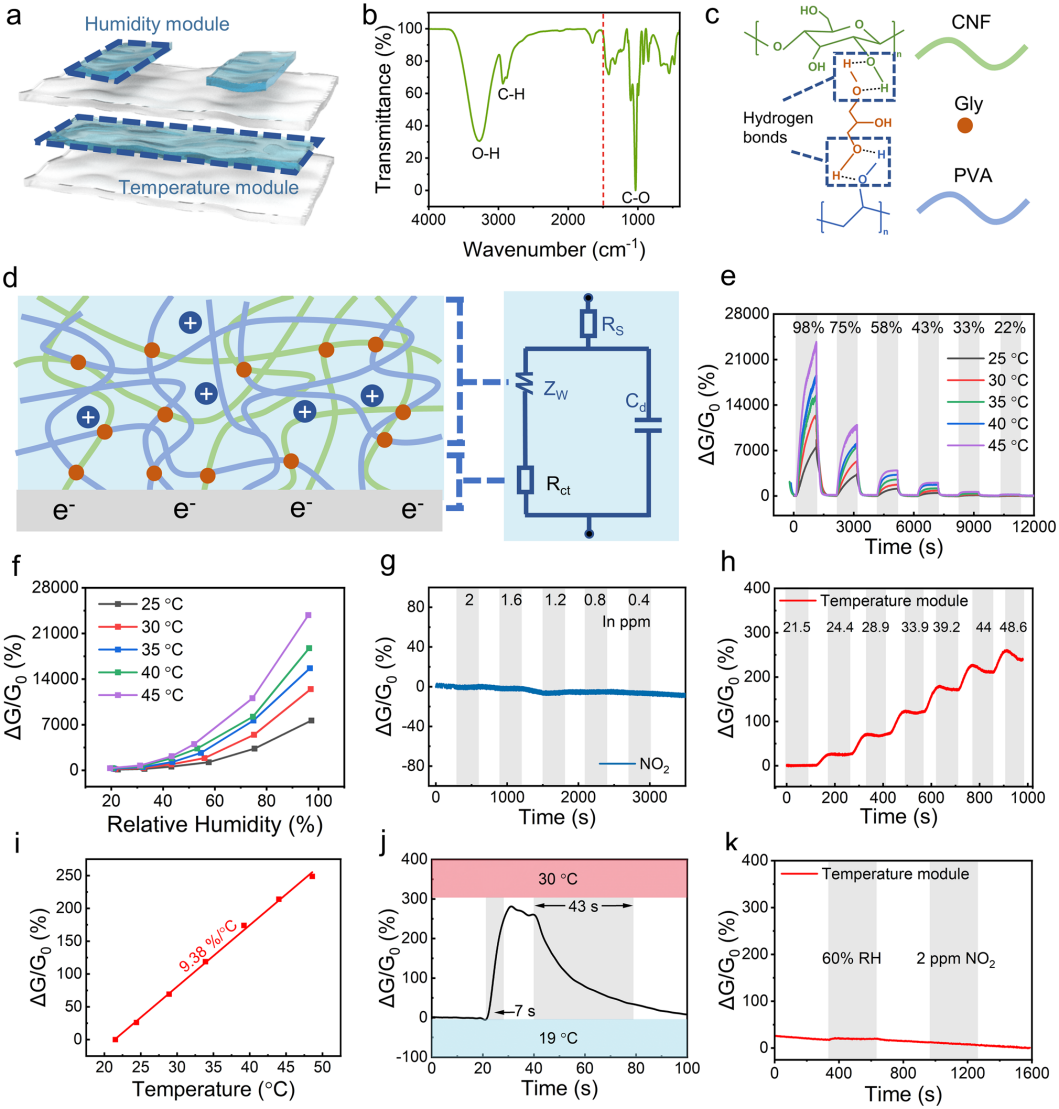
Humidity and temperature modules. **a** Multilayer stacked structure of the e-skin and the relative position of the humidity and temperature modules. **b** FTIR spectra of PVA-CNF organohydrogel. **c** PVA, Gly, and CNF molecules inside the PVA-CNF organohydrogel are bonded to each other through abundant hydrogen bonds. **d** Temperature and humidity sensing mechanisms and equivalent circuit fitting of PVA-CNF organohydrogel film. **e** Dynamic response curve of humidity module to 98%–22% RH at 25–45 °C. **f** Response changing curve of the humidity module versus relative humidity at 25–45 °C. **g** Dynamic response curve of humidity module to 2–0.4 ppm NO_2_, demonstrating its NO_2_-insensitivity. **h** Dynamic response curve of temperature module to 21.5–48.6 °C. **i** Response fitting curve of temperature module. **j** Response and recovery time analysis of the temperature module. **k** Dynamic response curve of temperature module to 60% RH and 2 ppm NO_2_, demonstrating its insensitivity to both NO_2_ and humidity

The humidity sensing module was fabricated by connecting Ag wires at both ends of the organohydrogel, and the temperature module was connected with conductive tapes and wrapped in Ecoflex to isolate humidity interference. Their relative positions in e-skin are shown in Fig. 2a. PVA-CNF organohydrogel contains a large amount of O–H inside, which can physically bond with free water molecules through hydrogen bonds. To verify this, Fourier transform infrared spectroscopy (FTIR) was performed on the PVA-CNF organohydrogel. It can be seen from the test results that there are three apparent characteristic peaks in the PVA-CNF organohydrogel (Fig. 2b). The C–H at 2940 cm^−1^ comes from the hydrocarbon backbone of the polymer chain, and the C-O at 1034 cm^−1^ mainly stems from the hydroxyl group and the ether bond on the CNF ring structure. Besides, the O–H at 3272 cm^−1^ originates from hydroxyl groups on the PVA chain, glycerol (Gly) molecules, and CNF. It is worth noting that there are three O–H bonds on a Gly molecule or CNF structural unit, which provides a large number of sites for the formation of hydrogen bonds, providing high water retention and good humidity sensitivity for the PVA-CNF organohydrogel. Thanks to the large number of O–H bonds, the molecular chains inside the PVA-CNF organohydrogel and the Gly molecules can be connected through hydrogen bonds to form a polymer network, which is consistent with the FTIR test results (Fig. 2c).

During the humidity sensing test, a 1 V and 200 Hz AC voltage was applied to both ends of the humidity module, and an LCR meter was used to record its resistance variation to detect relative humidity changes. The whole humidity module can be equivalent to a circuit consisting of resistors and capacitors (Fig. 2d) according to the electrochemical impedance spectroscopy for simplicity of analysis [59, 60]. *R*_s_ and *C*_d_ are the device’s inherent resistance and the electric double-layer capacitance, respectively. *R*_ct_ is the interfacial charge transfer resistance regulated by electrode reaction kinetics, and *Z*_W_ is the Warburg impedance affected by the diffusion process. Equations 1 and 2 are calculation formulas of the total impedance *Z* and the Warburg impedance *Z*_W_, respectively. In the low-frequency region, *R*_ct_ and *Z*_W_ dominate the total impedance of the device, and the real part of the total impedance can be simplified as Eq. 3. The *R*_ct_ and *Z*_W_ items in Eq. 3 show that the sensor’s resistance is dramatically affected by the charge transfer and mass diffusion process in the low-frequency region. As the humidity rises, the hydrogel absorbs water and swells, causing the unfolding of the internal polymer network. As a consequence, the hindering effect of the polymer network on the ion diffusion is weakened, manifested as the increase in diffusion coefficient ${\text{D}}_{{\text{H}}^{+}}$. Equations 3 and 4 indicate that the Warburg coefficient ($\sigma$) and the sensor’s resistance are inversely proportional to the diffusion coefficient [61]. An increase in ${\text{D}}_{{\text{H}}^{+}}$ eventually leads to a decrease in the sensor’s resistance. Besides, the continuous water absorption process results in more ionized protons and, at the same time, further promotes the charge transfer at the Ag/hydrogel interface, finally reducing the interface charge transfer resistance *R*_ct_. Under the synergistic effect of charge transfer and mass diffusion, the humidity module exhibits an increasing trend in conductance as the relative humidity rises.

1 $$\begin{array}{c}Z={R}_{\text{s}}+\frac{1}{j\omega {C}_{\text{d}}+\frac{1}{{R}_{\text{ct}}+{Z}_{\text{W}}}}\end{array}$$

2 $$\begin{array}{c}{Z}_{\text{W}}=\sigma {\omega }^{-\frac{1}{2}}\left(1-j\right)\end{array}$$

3 $$\begin{array}{c}{Z}_{Re}={R}_{\text{s}}+{R}_{\text{ct}}+\sigma {\omega }^{-\frac{1}{2}}\end{array}$$

Consider the special case of ${C}_{O}^{0}{D}_{O}^{1/2}={C}_{R}^{0}{D}_{R}^{1/2}$, then:

4 $$\begin{array}{c}\sigma =\frac{\text{RT}}{{\left(2D\right)}^{1/2}{\left(nF\right)}^{2}{C}^{0}} \end{array}$$

It can be seen from Eq. 4 that the Warburg coefficient is highly temperature-dependent. Therefore, the response of the humidity module at different temperatures needs to be calibrated. Figure 2e, f shows the response of the module to 22%–98% relative humidity at different temperatures. Due to the facilitation of temperature increment on interfacial charge transfer and mass diffusion processes, the module’s response to humidity increases with temperature. It can be seen from Fig. S4a that the humidity module shows superior response sensitivity in a more humid environment. Noticeably, the humidity response can be linearly fitted on the logarithmic coordinate, facilitating accurate measurement and calibration (Fig. S4b). In addition, since the humidity module is exposed to the external atmosphere, its response to gases is also worth studying. NO_2_ was selected as the target gas for subsequent measurement. During the testing process, the reactive ions in the electrolyte reciprocate under the drive of an alternating electric field. Due to the slow diffusion rate, there is not enough time for the reactive ions to move to the electrode side. Therefore, almost no electrochemical reaction occurs under alternating voltage. As shown in Fig. 2g, the humidity module has no response to NO_2_, which is consistent with the theoretical assumption.

During the rescue process, the injuries of trapped persons need to be confirmed timely to formulate a more specific rescue plan. Respiratory frequency, as an essential physiological indicator, can well reflect the physical condition of the injured. For respiratory monitoring, the multimodal e-skin with a high-performance humidity module was attached beneath the subject’s nostrils to record humidity changes caused by breathing (Fig. S5a). Figure S5b, c illustrates the dynamic conductance signal and response signal of the humidity module during respiration, where each subtle fluctuation in humidity corresponds to every breath taken. In order to eliminate baseline drift caused by rapid breathing, effective baseline correction methods need to be proposed (Fig. S5d). Spectrum analysis based on fast Fourier transform indicates that the effective signals are mainly distributed in the frequency band of 0.14–0.25 Hz (Fig. S5e). Thus, the finite impulse response (FIR) band-pass filter was adopted to screen out the effective frequency band and obtain a response signal with a flat baseline (Fig. S5f).

According to the previous analysis, humidity impacts interfacial charge transfer and material diffusion process and will finally change the resistance value of organohydrogel. Therefore, effective means to eliminate interference from humidity are essential for resistive temperature sensing modules. Inside the multimodal e-skin, the temperature sensing module is wrapped in waterproof Ecoflex to isolate humidity interference. Due to the Arrhenius relationship between *R*_ct_ or diffusion coefficient and temperature [62, 63], the temperature change will significantly affect the proton diffusion rate and the interfacial charge transfer rate, ultimately manifested as a change in the resistance value of the module. Figure 2h shows the dynamic response of the temperature module to 21.5–48.6 °C. According to the response fitting curve shown in Fig. 2i, the module exhibits a high temperature sensitivity of 9.38% °C^−1^. Experimental results indicate that the module possesses a linear response, a high temperature sensitivity and a positive temperature coefficient, which accords with theoretical analysis. It can be seen from Fig. 2j that the temperature module can respond promptly to temperature changes, with a response and recovery time of 7 and 43 s, respectively. In order to demonstrate the effect of Ecoflex packaging and AC voltage test methods on eliminating humidity and gas interference, the e-skin was placed in an environment with changing humidity and gas concentrations while recording the response signal of the temperature module. Figure 2k is the real-time dynamic response of the temperature module, which shows the module’s insensitivity to humidity and NO_2_.

### Pressure Module

**Fig. 3:**
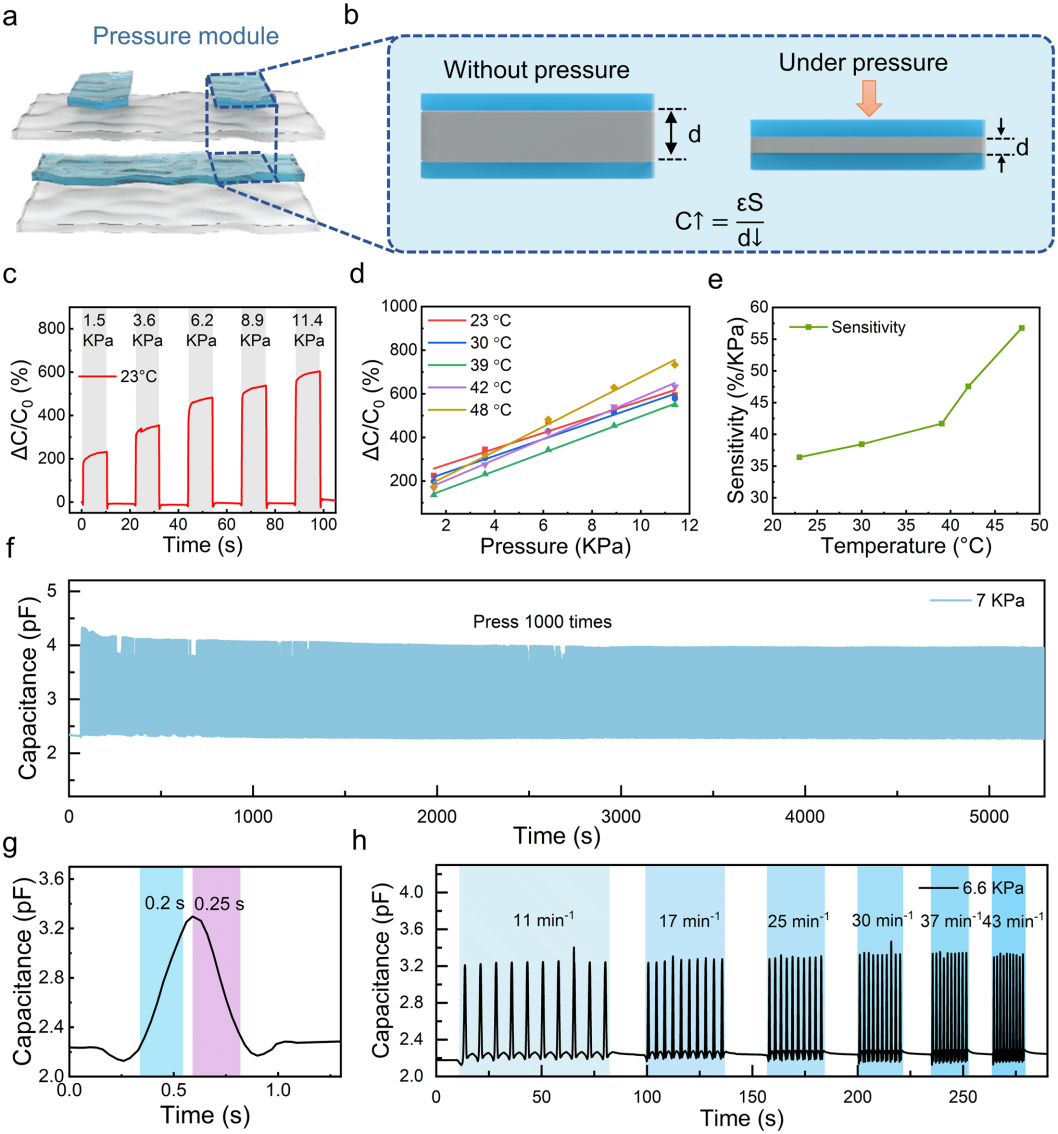
Pressure module. **a** Multilayer stacked structure of the e-skin and the relative position of the pressure module, which is composed of upper and lower gel electrodes and a sandwiched elastomer dielectric layer. **b** Schematic diagram of the sensing mechanism of the pressure module. **c** Real-time response signal of the pressure module under different pressures at 23 °C. **d** Pressure response fitting curves at different temperatures. **e** Sensitivity changes of pressure modules at different temperatures. **f** Recorded dynamic capacitance signal during 1000 times repeated pressure sensing test. **g** Response and recovery time analysis of pressure module. **h** Real-time response signals of the pressure module when subjected to pressure at different frequencies

The pressure sensing function of the e-skin is implemented with a sensitive parallel plate capacitor. Specifically, the top organohydrogel film and the middle Ecoflex-wrapped organohydrogel film are exploited as the capacitor’s upper and lower parallel plate electrodes, and Ecoflex is the dielectric between the plates (Fig. 3a). The distance between the upper and lower plates decreases when the module is squeezed (Fig. 3b). Increased stress will result in greater geometric deformation of the capacitor. According to Eq. 5, the reduction of the distance between the capacitor plates leads to an increase in the capacitance value. By recording the real-time capacitance signal of the module, accurate pressure measurement can be well-realized.

5 $$\begin{array}{c}C=\frac{\varepsilon S}{d}\end{array}$$

Figures 3c and S6 show the dynamic response signals of the pressure module when subjected to different pressures under different temperatures. It can be seen from Fig. 3d, e that the pressure sensitivity of the module increases as the temperature rises, which is attributed to the temperature-induced changes in the electrostatic properties of the material, such as the permittivity of the Ecoflex and conductivity of organohydrogel plates. According to Eq. 5, the capacitance of the pressure module is highly related to the relative permittivity of the Ecoflex dielectric layer. The enhanced effect of temperature on the dipole polarization leads to an increasing trend in the Ecoflex’s relative permittivity [64, 65]. Besides, the temperature-induced conductivity increase makes the charge distribution of the plate closer to an ideal conductor, equivalent to an increase in the effective area S. With larger relative permittivity and effective area, the module’s capacitance change (Δ*C*) is much more significant than the change of initial value (*C*_0_), resulting in a higher sensitivity at elevated temperatures. For studying the long-term performance stability, one thousand pressure cycling tests with a load pressure of 7 kPa were performed on the pressure module. As shown in Fig. 3f, the capacitance signal of the pressure module remained unchanged during the long-term test, demonstrating the module’s excellent long-term performance stability. Also, the pressure module shows a consistent pressure response signal in the pressure loading–unloading cycle test between 0.1 and 21.5 kPa (Fig. S7). In addition, the module has a swift response and recovery speed to pressure. As shown in Fig. 3g, the response and recovery time of the pressure module are 0.2 and 0.25 s, respectively. Owing to the module’s fast response and recovery characteristics, it can detect pressure step pulses with different frequencies (Fig. 3h).

### Proximity Module

**Fig. 4:**
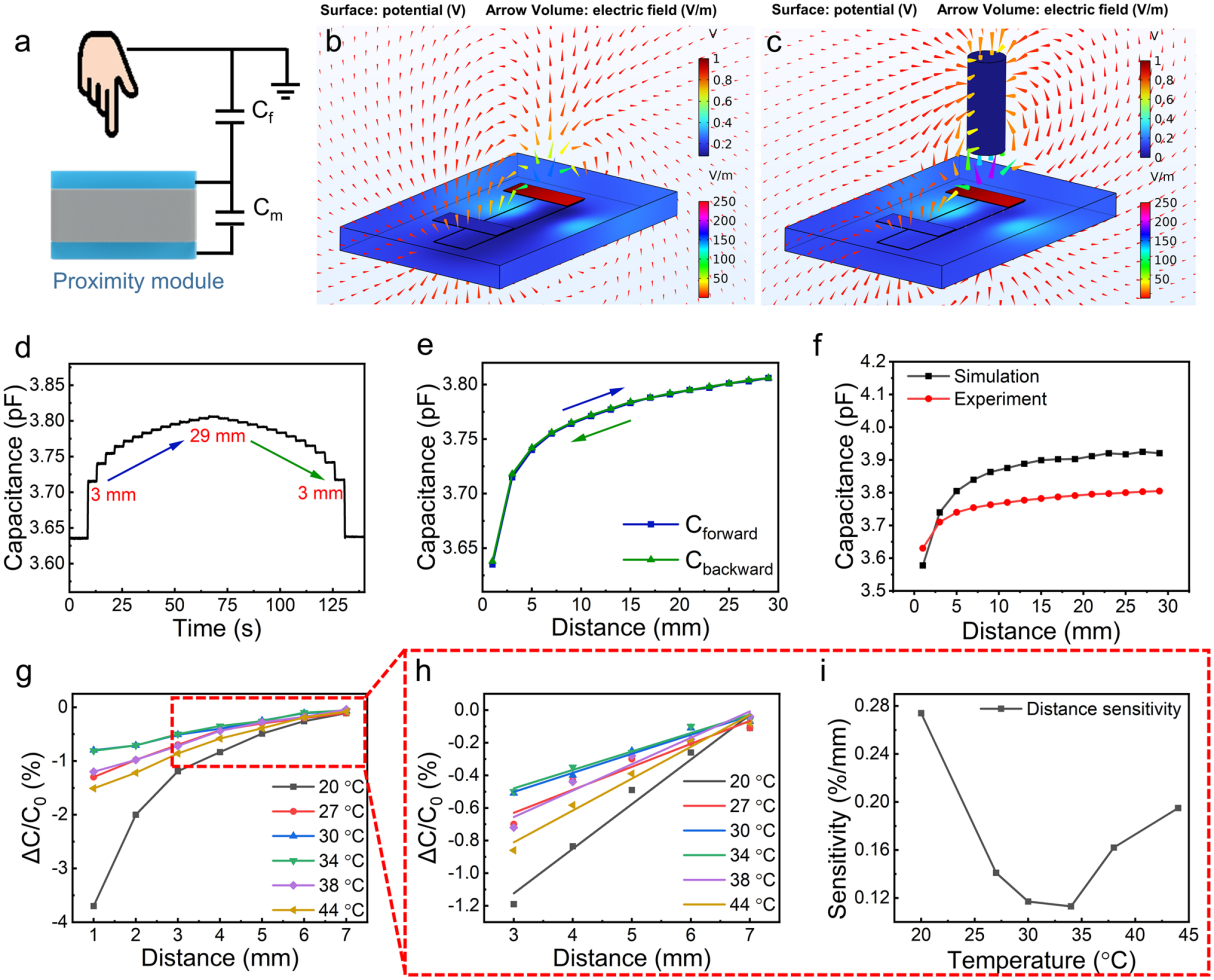
Proximity module. **a** Schematic diagram of the sensing mechanism of the proximity module. **b, c** FEM simulation images showing spatial electric field and potential distribution when the object is at an infinite distance and when the object approaches the proximity sensing module. **d** Real-time capacitance signal generated by the module when the distance between the object and the module increases from 3 to 29 mm and then decreases to 3 mm. **e** Capacitance-distance curve obtained when the distance between the object and the module increases from 3 to 29 mm and then decreases to 3 mm. **f** Comparison of the capacitance-distance curve obtained from FEM simulation and experiment. **g** Real-time response signal of the module when the distance between the object and the module increases from 1 to 7 mm under different temperatures. **h** The response fitting curve of the module when the distance is between 3 and 7 mm under different temperatures. **i** Sensitivity of proximity module under different temperatures

The top organohydrogel film and the Ecoflex-wrapped organohydrogel film are perpendicular to each other, forming a hydrogel-Ecoflex-hydrogel sandwich structure in the overlapping area for proximity sensing. The sensing mechanism of the proximity module can be explained as follows. The detection target can be regarded as a ground conductor during proximity sensing. When the detection target is at infinity, the electric field lines emitted by one of the organohydrogel films will terminate at the other one. As the detection object gradually approaches the sensing module, part of the electric field lines of the device pass through the object to the ground, eventually decreasing the equivalent capacitance of the capacitor *C*_m_. This can be equivalent to the process by which capacitor *C*_m_ charges capacitor *C*_f_, which is a capacitor formed with the object and the top layer of organohydrogel as electrodes and air as the dielectric (Fig. 4a). Analyzing from another perspective, when an object approaches the proximity module, part of the electric field lines emitting from the electrode plate flow into the ground, equivalent to a reduction in the plate area S. According to Eq. 5, the decreasing plate area S will lead to a decline in capacitance *C*_m_. To visually verify the proximity sensing mechanism, the proximity module was modeled using finite element analysis (FEM). An AC voltage with a frequency of 200 kHz and an amplitude of 1 V was applied between the upper and lower hydrogel plates. A conductive cylinder connected to the ground was used to simulate the target being detected. Since the charge relaxation time is much longer than the period of the driving voltage (5 $\upmu\text{s}$), the charges inside the proximity module cannot be regarded as stationary. Therefore, the current interface was chosen as the physics field for FEM analysis. It can be seen from Fig. 4b that when the object is at an infinite distance, the electric field lines emitted from one of the hydrogel plates flow entirely into the other one. As the object gradually approaches the proximity sensing module, some electric field lines flow into the ground through the metal cylinder, causing the capacitance to decrease (Fig. 4c). It is worth noting that the simulation result diagram shows that the organohydrogel surface potential is basically equal to 1 V with negligible voltage drop, which results from good conductivity and the small size of the organohydrogel film.

In the actual testing process, a self-made electric mobile platform was used to control the distance between the object and the proximity module. Firstly, the object was placed on the proximity module, and the distance between the object and the module was set as zero. Then, the object was gradually moved away to increase the distance between the object and the module. When the distance increased to 29 mm, the object was moved in the opposite direction to decrease the distance and finally returned to the initial position. The dwell time of the object at each position was 4 s. The real-time capacitance signal generated by the proximity module was recorded in Fig. 4d. It can be seen from Fig. 4e that the response-distance curves when the object is moved away from and approaches the module almost entirely coincide, indicating the proximity module’s performance consistency. Besides, the proximity module exhibits periodic dynamic response changes when the distance varies between 1 and 15 mm, also proving its excellent response consistency and performance stability (Fig. S8). The response-distance curves obtained from the experiment are very close to the simulation results, verifying the proximity sensing mechanism from another perspective and laying the foundation for future performance optimization (Fig. 4f). The slight difference between the simulation and experimental results mainly comes from the air domain boundary and limited precision meshing in FEM. Besides, the idealized approximation of the model and the disturbance of the electric field by surrounding objects during the actual test are also major causes of the error.

In order to study the influence of temperature on the sensing performance, the module was placed in different temperature environments for subsequent testing. The real-time response signal and response-distance curve are shown in Figs. S9 and 4g, respectively. Figure 4h is obtained by linear fitting the data points with a distance of 3–7 mm in Fig. 4g. When the temperature rises, the sensitivity of the proximity module first decreases and then increases, which is due to the trade-off between the conductivity of organohydrogel and the permittivity of Ecoflex dielectric layers (Fig. 4i). Due to the enhanced effect of temperature on the dipole polarization of Ecoflex, the relative permittivity of Ecoflex exhibits an increasing trend with rising temperature [64, 65]. According to Eq. 5, the increase in relative permittivity leads to an increasing device capacitance and makes it less susceptible to object proximity, which ultimately manifests as a decreased sensitivity. As the temperature continues to rise, the increase of organohydrogel’s conductivity begins to dominate the change in sensitivity. It can be derived from Gauss theorem that the voltage U between the plates is positively related to the effective charge on the plates *Q*_eff_ (Eq. 6). In the temperature rise process, the effective charge remains unchanged since both plates are driven by a voltage of 1 V. However, according to the electrostatic balance effect of the conductor, more charges trapped inside the plate will move to the surface when the conductivity increases with temperature. Consequently, the total charge Q required to maintain the driving voltage (or effective charge *Q*_eff_) decreases. As depicted in Eq. 7, the device capacitance shows a decreasing trend with the declining total charges and becomes more susceptible to object proximity, which ultimately manifests as an increased sensitivity.

6 $$\begin{array}{c}U=Ed=\frac{{\sigma }_{\text{eff}}d}{\varepsilon }=\frac{{Q}_{\text{eff}}d}{\varepsilon S}\end{array}$$

7 $$\begin{array}{c}C=\frac{Q}{U}\end{array}$$

### NO_2_ Module

**Fig. 5:**
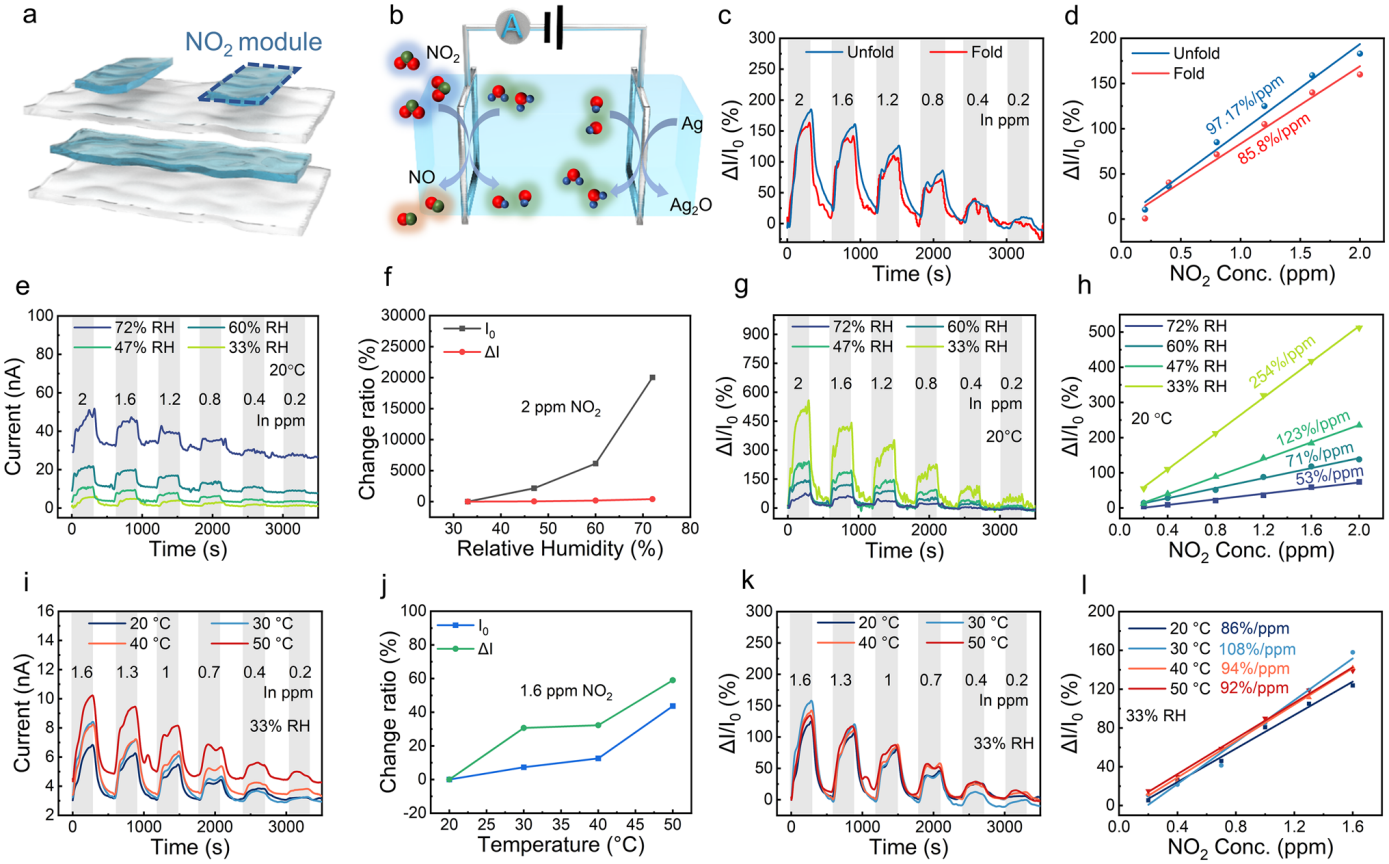
NO_2_ module. **a** Device structure of the NO_2_ module. **b** Schematic illustration of the electrochemical reaction-based sensing mechanism of the NO_2_ module. **c, d** Comparison of the dynamic response and sensitivity of the NO_2_ module in the complex folded and unfolded states, respectively. **e** Dynamic response current to 2–0.2 ppm NO_2_ under 33%–72% RH at 20 °C. **f** Plots of background current change ratio and response current change ratio to 2 ppm NO_2_ versus humidity at 20 °C. **g** Dynamic response curve to 2–0.2 ppm NO_2_ under 33%–72% RH at 20 °C. **h** Response fitting curve under 33%–72% RH at 20 °C. **i** Dynamic response current to 1.6–0.2 ppm NO_2_ under 20–50 °C at 33% RH. **j** Background current change ratio and response current change ratio to 1.6 ppm NO_2_ under 20–50 °C at 33% RH. **k** Dynamic response curve to 1.6–0.2 ppm NO_2_ under 20–50 °C at 33% RH. **l** Response fitting curve of NO_2_ module versus NO_2_ concentration under 20–50 °C at 33% RH

Multimodal e-skin based on organohydrogel not only possesses the temperature, humidity, and pressure perception capabilities of natural skin but also develops a function beyond it, that is, the perception of toxic gas. The device structure and sensing mechanism of the NO_2_ module are shown in Fig. 5a, b, respectively. Both ends of the top PVA-CNF hydrogel film were connected with Ag wires to form a typical electrochemical gas sensor with Ag as electrodes and hydrogel as solid electrolytes. At the cathode, NO_2_ is reduced to NO, generating a Faradaic current proportional to the NO_2_ concentration (Eq. 8). At the anode, Ag is oxidized to Ag_2_O, as shown in Eq. 9. To demonstrate the flexibility of e-skin, the folded and unfolded multimodal e-skins were used to measure the dynamic gas response under different NO_2_ concentrations (Figs. 5c and S10). Figure 5c, d shows that the NO_2_ module has highly consistent response signals in the folded and unfolded states, which ascribes to the relatively fixed mass transfer distance between the two electrodes.

8 $$\begin{array}{c}\text{N}{\text{O}}_{2}+{\text{H}}_{2}\text{O}+2{\text{e}}^{-}\to \text{NO}+{2\text{OH}}^{-}\end{array}$$

9 $$\begin{array}{c}2\text{Ag}+2{\text{OH}}^{-}-2{\text{e}}^{-}\to {\text{Ag}}_{2}\text{O}+{\text{H}}_{2}\text{O}\end{array}$$

It is worth noting that since H_2_O participates in the cathode reaction, the NO_2_ response of the room-temperature sensor is inevitably affected by the change in ambient humidity. Besides, temperature is also an essential factor affecting the electrochemical reaction rate. In order to study the temperature and humidity dependence of the NO_2_ module, the multimodal e-skin was placed in different humidity or temperature environments for NO_2_ testing.

According to Eq. 8, elevated ambient relative humidity will promote the reduction reaction and eventually increase the response current, which is consistent with the experimental results in Fig. 5e. Since the thinned hydrogel material is susceptible to ambient humidity, a slight increase in ambient humidity will lead to a dramatic rise in the baseline current. It can be seen from Figs. S11a and 5f that despite the comparable change amplitude of the background current and response current, the change ratio of the background current is much higher than that of the response current as the relative humidity increases. When the relative humidity increased from 33% to 72% RH, the background current changed by 20,039%, while the response current only increased by about 400%. Therefore, both the response value and response sensitivity of the NO_2_ module decrease as the relative humidity increases (Figs. 5g, h and S11b).

In addition to humidity, temperature is also an essential factor affecting the performance of the NO_2_ module. It can be seen from Fig. 5i that both the NO_2_ response current and the background current of the NO_2_ sensing module increase with the temperature. The increase in the response current is attributed to the promotion of temperature on the electrochemical reaction rate, while the background current drift is due to the change in the conductivity of the hydrogel film caused by the temperature rise. When the ambient temperature increased from 20 to 50 °C, the background current and the response current have comparable change amplitudes and ratios (Figs. S11c and 5j). Therefore, even under different temperature conditions, the response performance of the NO_2_ module exhibits limited change in response and sensitivity (Figs. S11d and 5k, l). Based on the sensitivity and background noise level, the theoretical limit of detection (LOD) of the NO_2_ module is calculated as 11.1 ppb (Fig. S12; Table S5). The extremely low LOD of the NO_2_ module enables the e-skin to monitor ppb-level NO_2_ in the environment. In order to characterize the performance repeatability of the NO_2_ module, the entire device was placed in an environment with alternating cycles of N_2_ and 0.8 ppm NO_2_. As can be seen from Fig. S13, the NO_2_ module shows good performance consistency over five cycles. Compared with the representative NO_2_ sensors reported so far, the NO_2_ module using PVA-CNF organohydrogel as the sensitive material has good stretchability and exhibits superior sensing performance with higher sensitivity (254% ppm^−1^) and a low detection limit (11.1 ppb) at room temperature (Table S6) [66–72]. Most importantly, the NO_2_ sensing module can perform response calibration through the integrated temperature and humidity module in the multimodal e-skin, thereby accurately monitoring NO_2_ even in application scenarios with dramatic temperature and humidity changes.

### Multisensory Robot Hand for Object Recognition

**Fig. 6:**
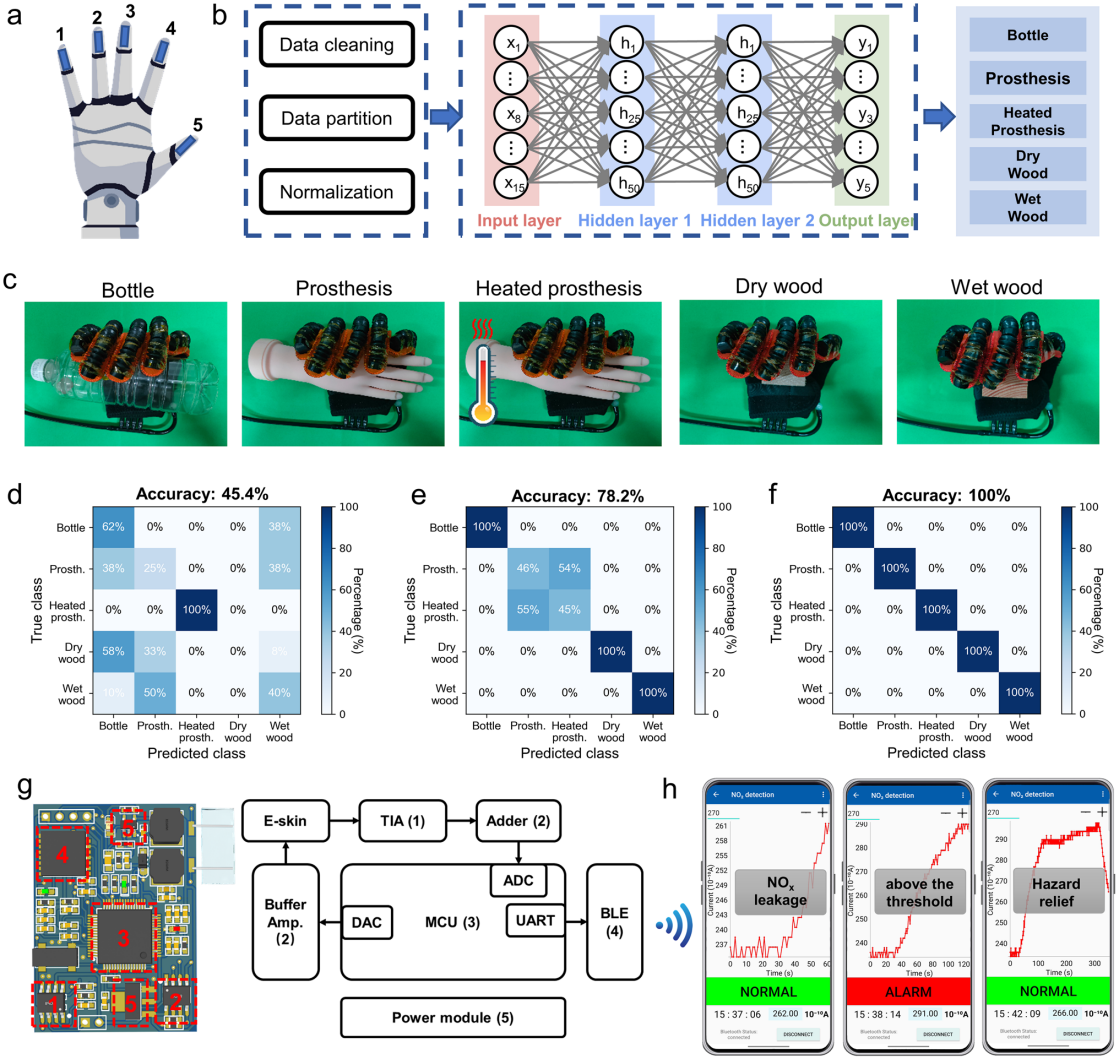
Multimodal e-skin for environmental object recognition and toxic gas alarm. **a** Schematic diagram of the robot hand integrated with five multimodal e-skins. **b** Flow chart of data preprocessing and the DNN model structure for identifying objects. **c** Photographs showing the robot hand grasps five different objects, including plastic bottles, prostheses, heated prostheses, dry wood, and wet wood. **d** Classification test confusion matrix when using only temperature modules of multimodal e-skin, **e** using pressure & proximity modules and humidity modules of multimodal e-skin and **f** using pressure & proximity, temperature, and humidity modules of multimodal e-skin. **g** Physical diagram and system block diagram of the integrated NO_x_ wireless monitoring system. **h** Real-time monitoring and alarm of leak incidents on a smartphone using our integrated NO_x_ wireless monitoring system

The hardware circuit can be an interface that converts the sensor’s physical signal into a digital signal, while the machine learning algorithm can use the collected data to decode information and provide behavioral guidance for users [73, 74]. In order to demonstrate the feasibility of applying machine-learning assisted multimodal e-skin for robot perception and post-earthquake rescue, the devices were installed on the ends of the five fingers of the pneumatic glove, simulating the soft robot hand to grasp and identify objects with the assistance of AI (Fig. 6a, b). The robot hand equipped with e-skin repeatedly grabbed plastic bottles, rubber prosthetic hands, heated rubber prosthetic hands, dry wood, and wet wood (Fig. 6c). Heated prosthetic hands were used to simulate exposed body parts of trapped persons. The average value of extracted data was normalized and displayed as a series of heatmaps, thereby visualizing the proximity/pressure, temperature and humidity distribution when the robot hand grasps different objects (Fig. S14). It is worth noting that both the response signals of the pressure and proximity modules are expressed as changes in capacitance values. When the robotic fingers approach the object without pressing, the capacitance response signal originates from changes in proximity, while when the robot hand fingers contact and press the object, the pressure on the e-skin will change the capacitance value of the pressure module. The flow chart of data preprocessing, model construction and training, and object recognition is summarized and displayed in Fig. 6b**.** Firstly, the temperature, humidity, and pressure/proximity response signals collected by the e-skin were extracted and divided into training sets and test sets with a ratio of 1:1 after data cleaning. In order to make model training converge faster, Z-score normalization was performed on the 15 features of the data. After data preprocessing, the training set was input into the constructed DNN model and trained iteratively for 200 epochs. The test set was used to evaluate the generalization ability of the model.

Compared with single-modal or dual-modal e-skins, rescue robots integrated with multimodal e-skins can obtain more types of information while grasping objects, dramatically improving the recognition accuracy and generalization ability of the DNN model. As depicted in Fig. 6d, with only the temperature sensing function, the rescue robot cannot distinguish objects except heated prostheses, exhibiting a low recognition accuracy (45.4%). Similarly, when only the pressure module or humidity module exists, the recognition accuracy of the rescue robot is 50.2% and 46.8%, respectively (Fig. S15a, b). When the humidity module is blocked, the rescue robot cannot distinguish between dry and wet wood blocks. The total recognition accuracy using only pressure/proximity sensing and temperature sensing reaches about 82.8%, as shown in Fig. S15c. When the temperature module was missing, the rescue robot performed poorly in classifying prostheses and heated prostheses and only had an accuracy of 78.2% (Fig. 6e). The combination of pressure/proximity, temperature and humidity sensing of 5 e-skins can improve the recognition accuracy and completely discriminate five different objects, with an accuracy of 100% (Fig. 6f). The change curves of the loss function and accuracy with the number of iterations during the DNN model training process are shown in Fig. S16. It is noticeable that the loss function and accuracy remain stable after 40 iterations, demonstrating the rapid convergence of the model. Compared with single-modal or dual-modal e-skin, multimodal e-skin endows the robot with more powerful multisensory and anti-interference capabilities, which can significantly improve its object recognition accuracy. Robot perception is an indispensable part of robot application. The emergence of multimodal e-skin has dramatically broadened the application boundaries of machine automation. Robots with multisensory capabilities are expected to perform various complex tasks, such as accurately identifying human bodies and performing post-earthquake rescue missions in dangerous environments.

### Wireless Monitoring of NO_2_ Concentrations and Prompt Leak Alarms

Severe earthquake disasters may damage the production equipment and storage tanks of chemical companies, leading to leaks of hazardous chemicals and environmental pollution incidents. Nitrogen oxides, as typical toxic gases in industrial production [48], can cause severe damage to the human respiratory system and nerve center once inhaled [49, 50], the leakage of which may bring difficulties to rescue missions at earthquake disaster sites. By equipping the integrated NO_x_ wireless monitoring system that combines multimodal e-skin with wireless gas alarm circuits, the rescue robot can be endowed with the function of real-time monitoring toxic gases at earthquake disaster sites, thereby taking appropriate rescue measures to help trapped people escape from the toxic environment.

Figure 6g is a physical diagram and system block diagram of the integrated NO_x_ wireless monitoring system. The wireless alarm circuit is controlled by the STM32F103RCT6 microprocessor, in which a 12-bit digital-to-analog converter (DAC) is used to output a voltage of 0.5 V. The DAC output voltage is connected to a unit-gain buffer amplifier to ensure sufficient driving capability for the next-stage NO_2_ module. The module’s response current is converted into a voltage signal through a trans-impedance amplifier (TIA). Subsequently, the voltage signal is adjusted to a suitable voltage range by an adder and then read by the microcontroller unit (MCU) through a 12-bit analog-to-digital converter (ADC). The recorded data is processed by the arithmetic mean filtering in the MCU to filter out noise signals and is converted back into the corresponding current data. Finally, the processed data is sent to the Bluetooth low-energy (BLE) chip through UART, and the BLE module wirelessly transmits the data to the mobile terminal using a specific protocol for further processing.

The entire device was placed in a closed environment with controllable gas composition, and 1 ppm NO_2_ was injected into the closed gas chamber to simulate a NO_x_ leak event. The real-time current signal collected and processed by the integrated NO_x_ monitoring system was wirelessly transmitted to the self-programming App on the mobile phone. As can be seen from Fig. 6h, when NO_x_ gas leakage occurred, the current value displayed by the App increased rapidly, indicating a rise in the concentration of NO_x_ in the environment. When the current value was higher than the set current threshold (27 nA), the App alerted the user of excessive NO_x_ concentration in the environment, which may endanger users’ safety. Finally, pure nitrogen was injected into the closed air chamber to purge NO_x_. As the NO_x_ concentration dropped below the threshold, the NO_x_ alarm signal on the mobile App was released. The above experiments demonstrate the potential of the integrated NO_x_ wireless monitoring system for remote NO_x_ monitoring and prompt leak alarms. The combination of multimodal e-skin and wireless alarm circuits greatly expands the environmental perception capabilities of rescue robots and provides strong technical support for the development of various complex rescue missions.

## Conclusions

In summary, a multimodal e-skin with excellent temperature, humidity, pressure, proximity, and NO_x_ gas sensing capabilities was proposed for the first time. The e-skin, which is stacked by multiple layers of flexible materials of Ecoflex and PVA-CNF organohydrogel, has not only flexibility, stretchability and sensation similar to natural skin but also the sensing function beyond natural skin-detecting the proximity of objects and extremely low NO_2_ concentration. Noticeably, the e-skin integrated with temperature and humidity modules can monitor ambient temperature and humidity changes, calibrate the response curves of other modules, and avoid the impact of external factors on the sensor accuracy. The e-skin based on sensitive material of PVA-CNF organohydrogel exhibits fast pressure response time (0.2 s), high temperature sensitivity (9.38% °C^−1^), a wide range of humidity response (22%–98% RH), a low gas detection limit (11.1 ppb), high NO_2_ sensitivity (254% ppm^−1^) and the ability to perceive the proximity of objects accurately. Integrating multimodal e-skin on the rescue robot endows it with the multi-perception of the environment. The combination of artificial intelligence algorithms and multimodal e-skin can realize the accurate classification of environmental objects and identify human limbs from them, thus significantly improving the rescue robot’s search and rescue efficiency in earthquake ruins. Compared with single-modal or dual-modal e-skin, multimodal e-skin can obtain more types of information in one grasping action, thereby improving the accuracy and generalization ability of the DNN model, achieving an accuracy improvement from 45.4% to 100%. In addition, by combining the e-skin with the NO_2_ wireless alarm circuit, the rescue robot can monitor the toxic gas in the environment in real-time and wirelessly transmit the data to the remote terminal through low-power Bluetooth technology. Based on the data received, the rescue center can capture the changes in the gas environment at the earthquake site and respond quickly to NO_x_ leak incidents. In this work, the e-skin with multi-sensing performance beyond natural skin constructs an interface for intelligent robots to interact with the physical world. With the synergy of artificial intelligence algorithms and hardware circuits, robots integrated with multimodal e-skin show more powerful information processing, decoding, and transmission capabilities and are expected to be applied to more diversified and challenging scenarios.

## Supplementary Information

Below is the link to the electronic supplementary material.

Supplementary file1 (DOCX 7193 KB)

Supplementary file2 (MP4 10173 KB)
