# Supplementary material for: Design of AI-Enhanced and Hardware-Supported Multimodal E-Skin for Environmental Object Recognition and Wireless Toxic Gas Alarm
Source: Nanomicro Lett. 2024 Jul 29;16:256. doi: 10.1007/s40820-024-01466-6 (PMC11286924; doi:10.1007/s40820-024-01466-6)
Supplement: Supplementary file 1 — Supplementary file1 (DOCX 7193 KB) [file 40820_2024_1466_MOESM1_ESM.docx]

Supporting Information for

**Design of AI-Enhanced and Hardware-Supported Multimodal E-Skin for Environmental Object Recognition and Wireless Toxic Gas Alarm**

Jianye Li^1,6^, Hao Wang^1^, Yibing Luo^1^, Zijing Zhou^1^, He Zhang^7^, Huizhi Chen^9,10^, Kai Tao ^2,3,^*, Chuan Liu^1^, Lingxing Zeng^11^, Fengwei Huo^4,5^* and Jin Wu^1,6,7,8,^*

^1^State Key Laboratory of Optoelectronic Materials and Technologies and the Guangdong Province Key Laboratory of Display Material and Technology, School of Electronics and Information Technology, Sun Yat-Sen University, Guangzhou, 510275, People’s Republic of China

^2^Ministry of Education Key Laboratory of Micro and Nano Systems for Aerospace, School of Mechanical Engineering, Northwestern Polytechnical University, Xi'an, 710072, People’s Republic of China

^3^Research & Development Institute of Northwestern Polytechnical University in Shenzhen, Shenzhen, 518063, People’s Republic of China

^4^The Institute of Flexible Electronics (IFE, Future Technologies), Xiamen University, Xiamen, 361005, People’s Republic of China

^5^Key Laboratory of Flexible Electronics (KLOFE), School of Flexible Electronics (Future Technologies), Nanjing Tech University, 30 South Puzhu Road, Nanjing, 211816, People’s Republic of China

^6^State Key Laboratory of Transducer Technology, Shanghai, 200050, People’s Republic of China

^7^Guangdong Provincial Key Laboratory of Technique and Equipment for Macromolecular Advanced Manufacturing, Guangzhou, 510641, People’s Republic of China

^8^State Key Laboratory of Polymer Materials Engineering, Sichuan University, Chengdu, 610065, People’s Republic of China

^9^Guangdong Provincial Key Laboratory of Research and Development of Natural Drugs, and School of Pharmacy, Guangdong Medical University, Dongguan, 523808, People’s Republic of China

^10^The First Dongguan Affiliated Hospital, Guangdong Medical University, Dongguan, 523808, People’s Republic of China

^11^Engineering Research Center of Polymer Green Recycling of Ministry of Education, College of Environment and Resources, Fujian Normal University, Fuzhou, Fujian, 350007, People’s Republic of China

*Corresponding authors. E-mail: [wujin8@mail.sysu.edu.cn](mailto:wujin8@mail.sysu.edu.cn) (Jin Wu); [taokai@nwpu.edu.cn](mailto:taokai@nwpu.edu.cn) (Kai Tao); [iamfwhuo@njtech.edu.cn](mailto:iamfwhuo@njtech.edu.cn) (Fengwei Huo)

**Supplementary Figures and Tables**


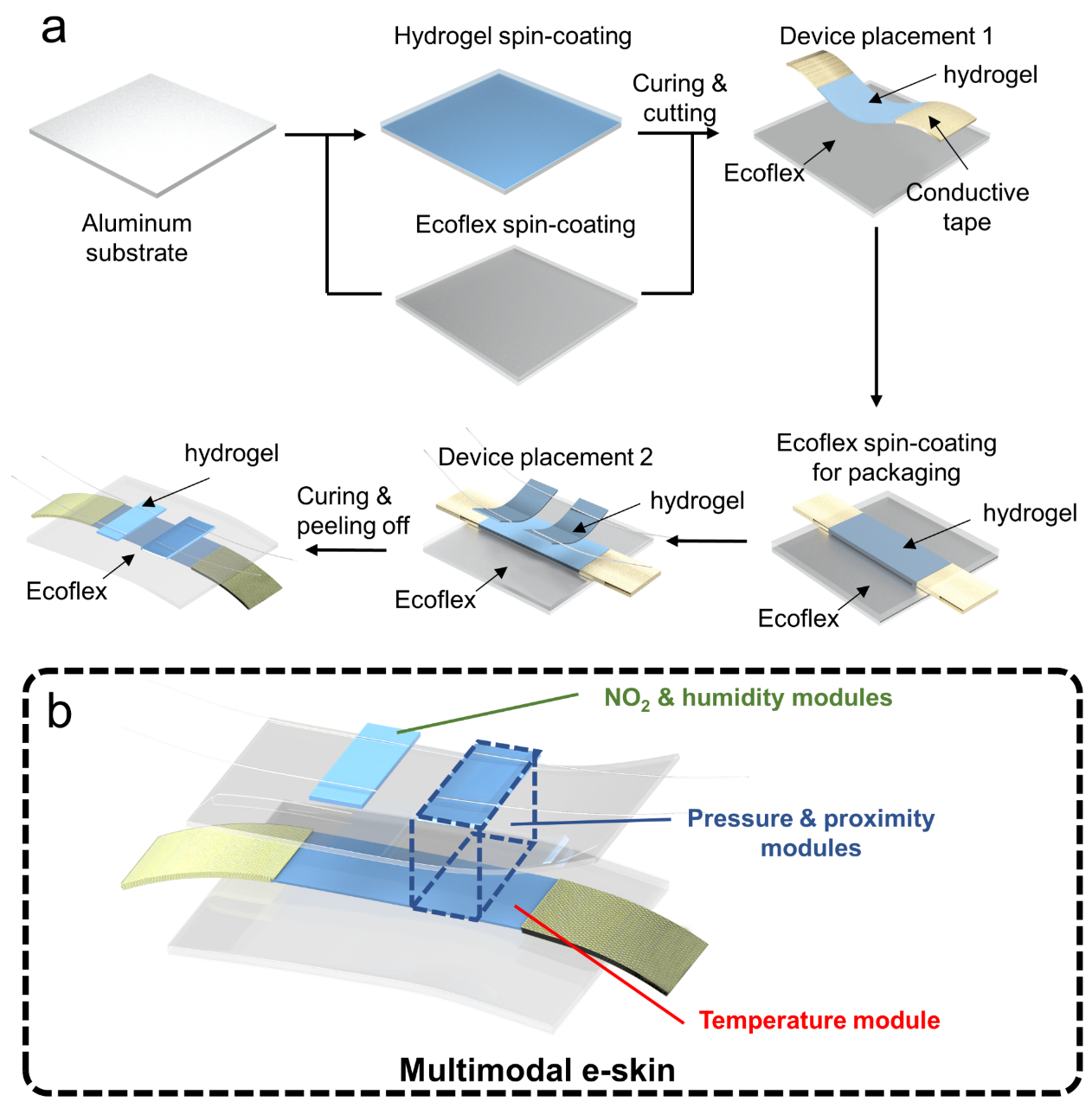


**Fig. S1** Flowchart for the fabrication of multimodal e-skin and schematic diagram showing the relative positions of different sensing modules. **a** Firstly, the hydrogel and the Ecoflex precursors were spin-coated on alumin um substrates and placed at room temperature for curing. After curing, the organohydrogel film was cut to the appropriate size and connected with conductive tape to construct the conductive temperature sensor. The temperature module was then placed on top of the cured Ecoflex and covered by another layer of Ecoflex through spin-coating. Two hydrogel films with suitable sizes were connected with silver wires and placed on the uncured Ecoflex layer. The entire device was kept at room temperature until the top Ecoflex layer was cured. Finally, the whole device was peeled off from the aluminum substrate to obtain a multimode e-skin. **b** Schematic diagram showing the relative positions of five different sensing modules


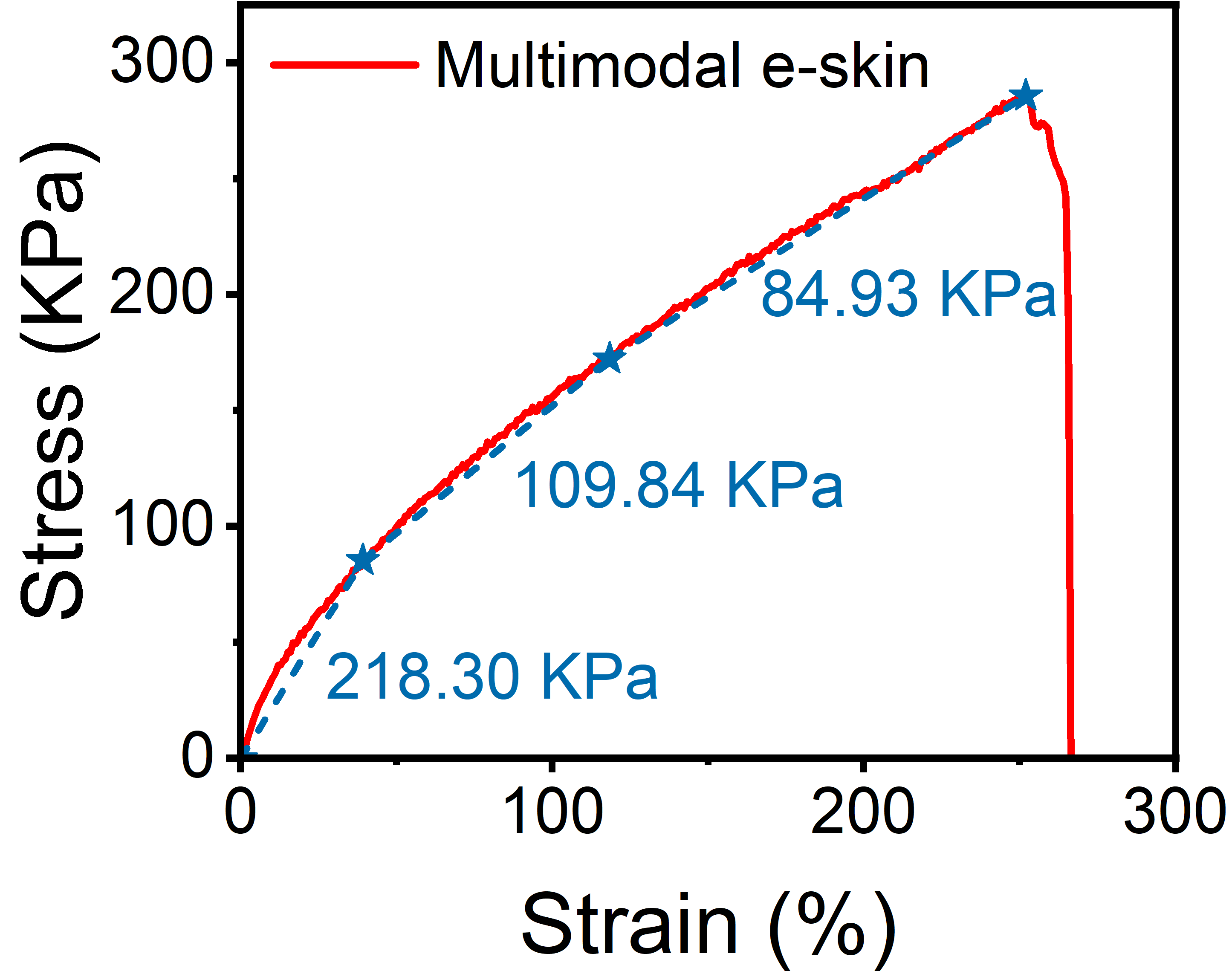


**Fig. S2** Stress-strain curve of multimodal e-skin


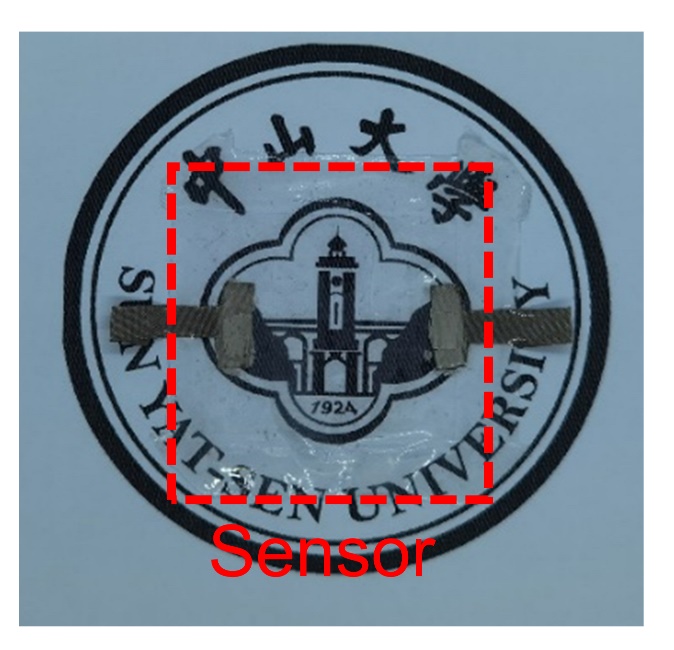


**Fig. S3** University logo pattern below the multimodal e-skin is visible, demonstrating its excellent transparency


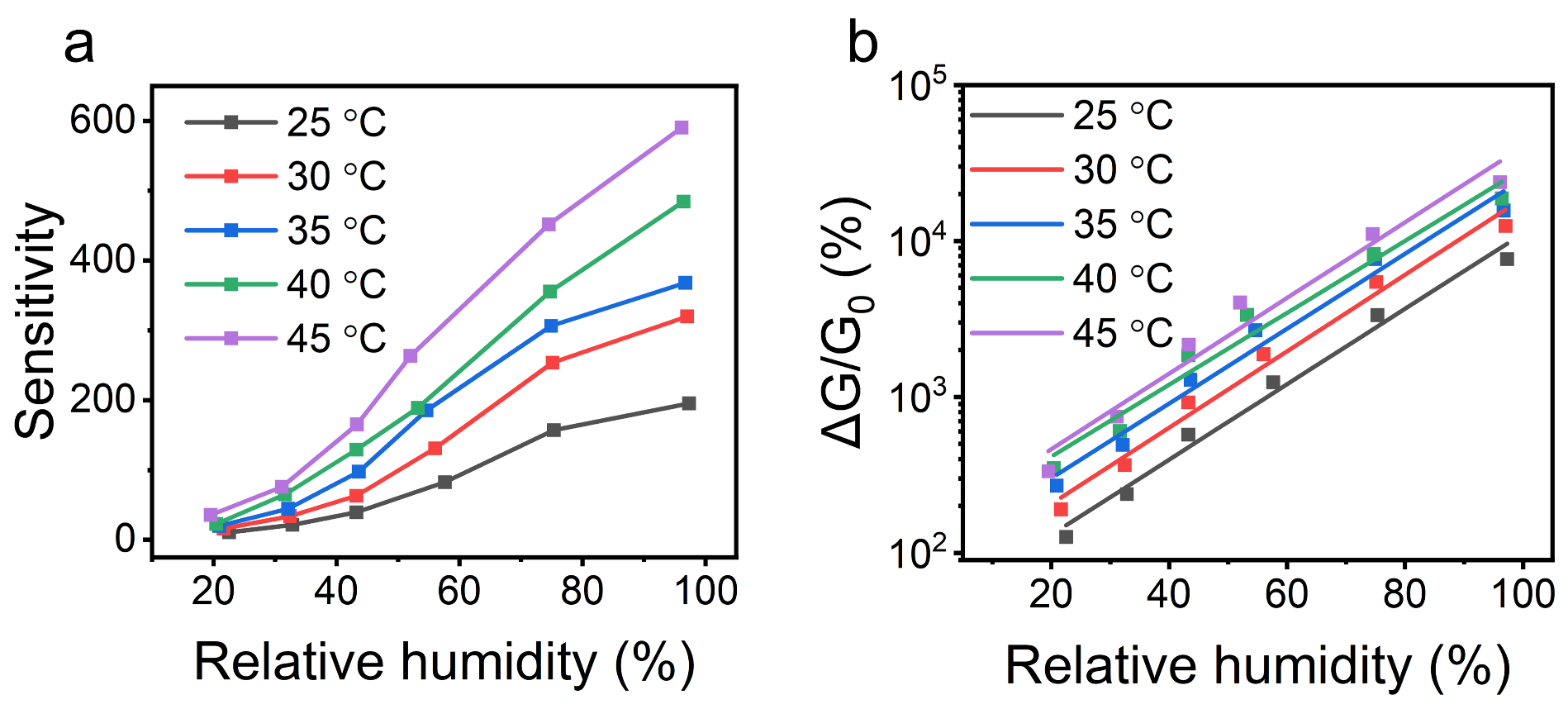


**Fig. S4** At different temperatures, the sensitivity-humidity change curve and the response fitting curve of the humidity module. **a** Sensitivity-RH curves of the humidity module at different temperatures. **b** On the logarithmic scale, the humidity response is linearly related to the relative humidity


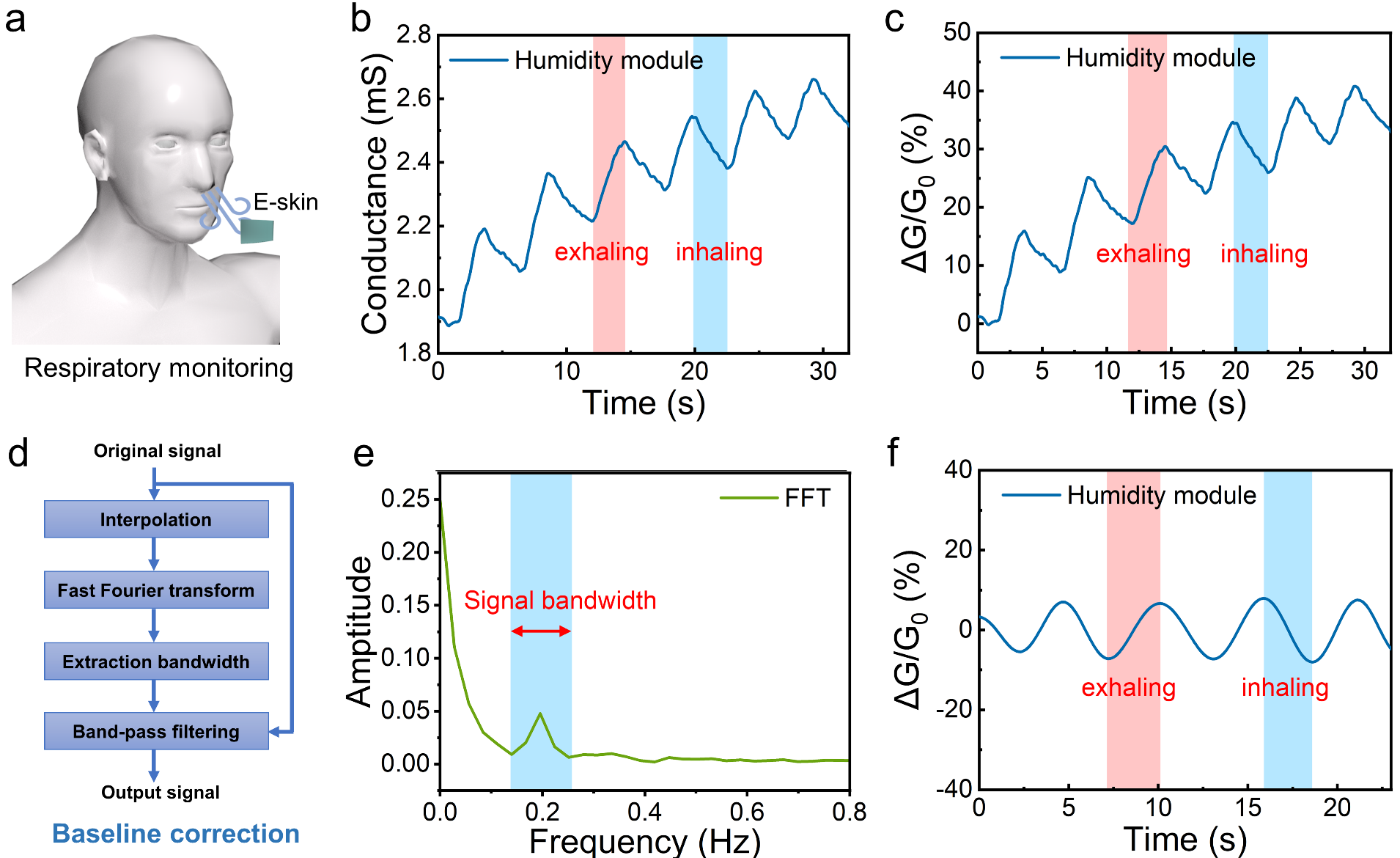


**Fig. S5** Respiratory monitoring based on multimodal e-skin. **a** Schematic illustration of the multimodal e-skin monitoring nasal respiratory rate through detecting changes in humidity. **b, c** Real-time conductance and response signal recorded by the humidity module during the subject's breathing. **d** Flow diagram of baseline correction. **e** Spectral diagram of the original humidity response signal obtained by fast Fourier transform. **f** Real-time humidity response after baseline correction through FIR band-pass filtering.

**The detailed code about the baseline correction ran on MATLAB R2022b and is presented as follows:**

P2_1 = P2_2(1:N/2+1);

P2_1(2:end-1) = 2*P2_1(2:end-1);

f = Fs*(0:(N/2))/N;

subplot(4,1,1);

plot(x1,y1);

title('Original signal');

subplot(4,1,2);

plot(f,P1_1);

title('FFT of original signal');

subplot(4,1,3);

plot(x1,y2);

title('Output signal');

subplot(4,1,4);

plot(f,P2_1);

title('FFT of output signal');

N=358;

Fs=10;

x1=0:0.1:35.7;

% Data interpolation

y1=interp1(x,y,x1, 'pchip');

% Band-pass filtering

y2 = 45*filter(h',1,y1);

% Fast Fourier transform

Y1 = fft(y1);

Y2 = fft(y2);

% Plotting

P1_2 = abs(Y1/N);

P1_1 = P1_2(1:N/2+1);

P1_1(2:end-1) = 2*P1_1(2:end-1);

P2_2 = abs(Y2/N);


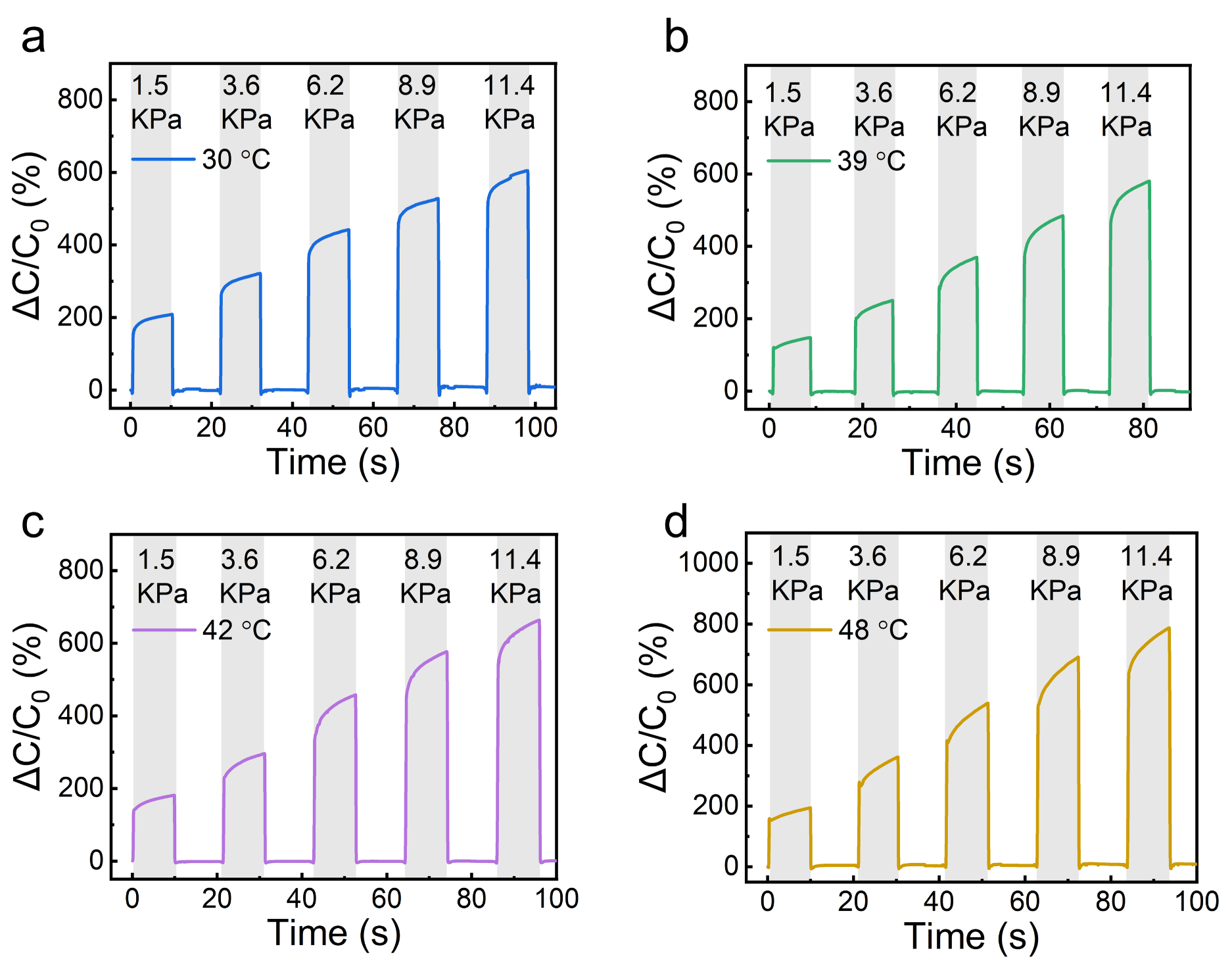


**Fig. S6** Real-time response signals of the pressure module to different pressures at 30, 39, 42, and 48 ℃


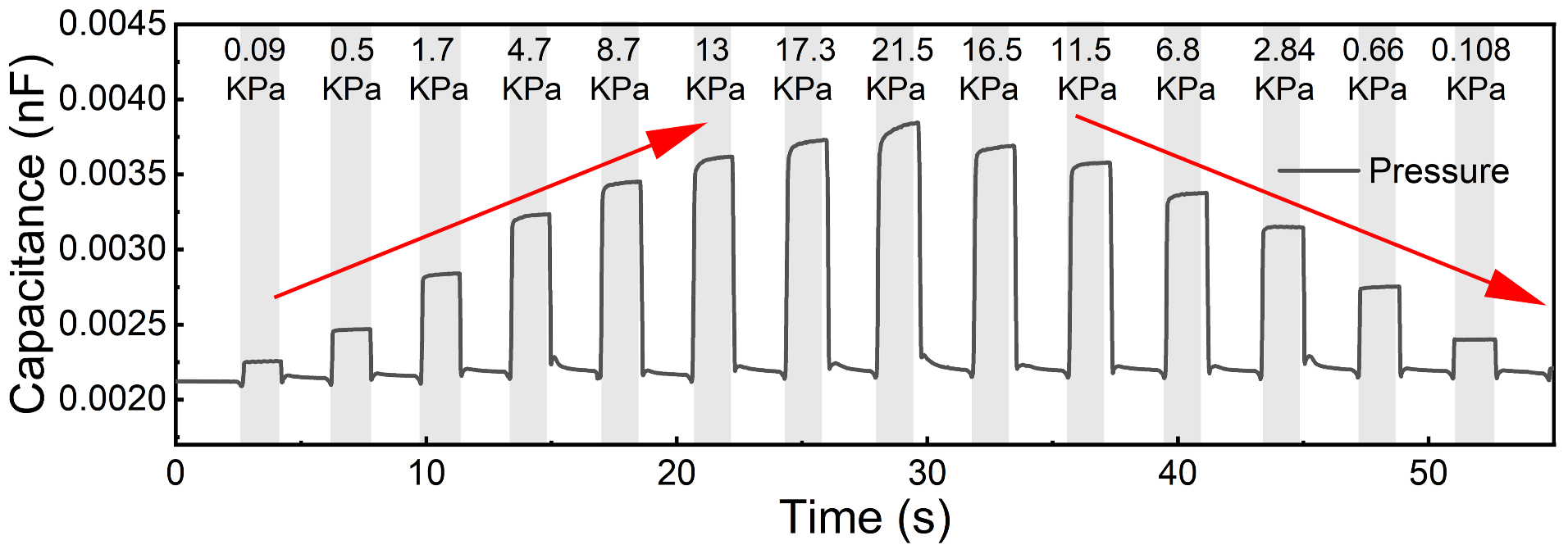


**Fig. S7** Real-time capacitance signal change of pressure module during cyclic loading-unloading test of different pressures


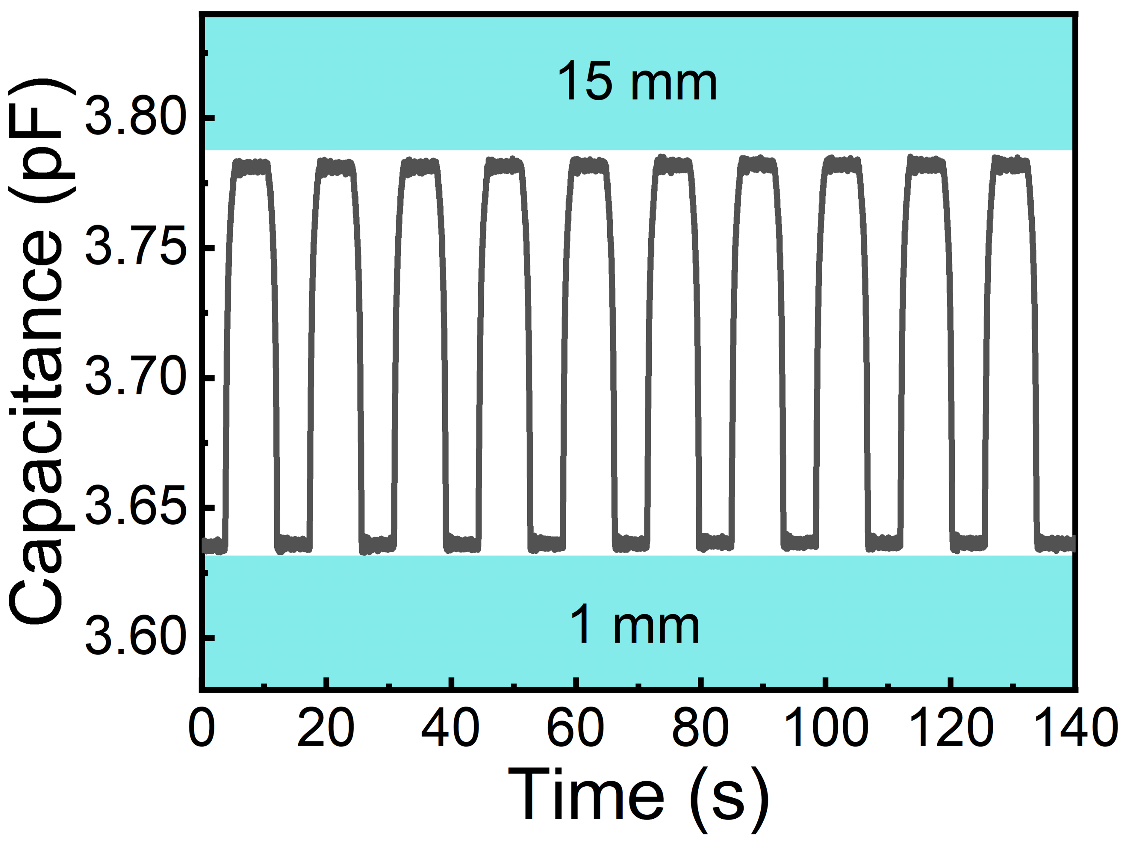


**Fig. S8** Dynamic response signal of the proximity module when the distance varies between 1 and 15 mm


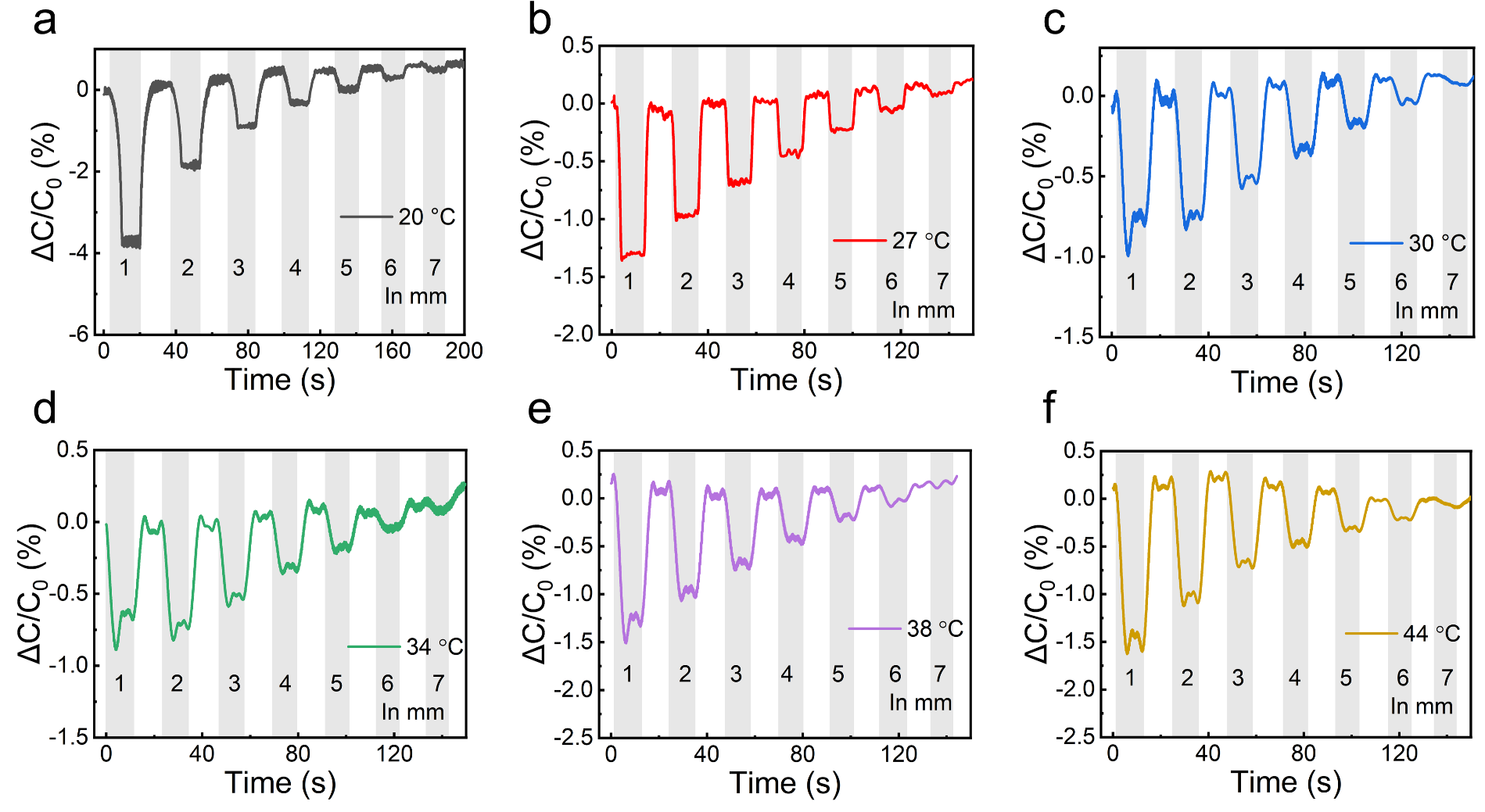


**Fig. S9** Real-time response signals of the proximity module when the object gradually approaches and leaves under different temperatures


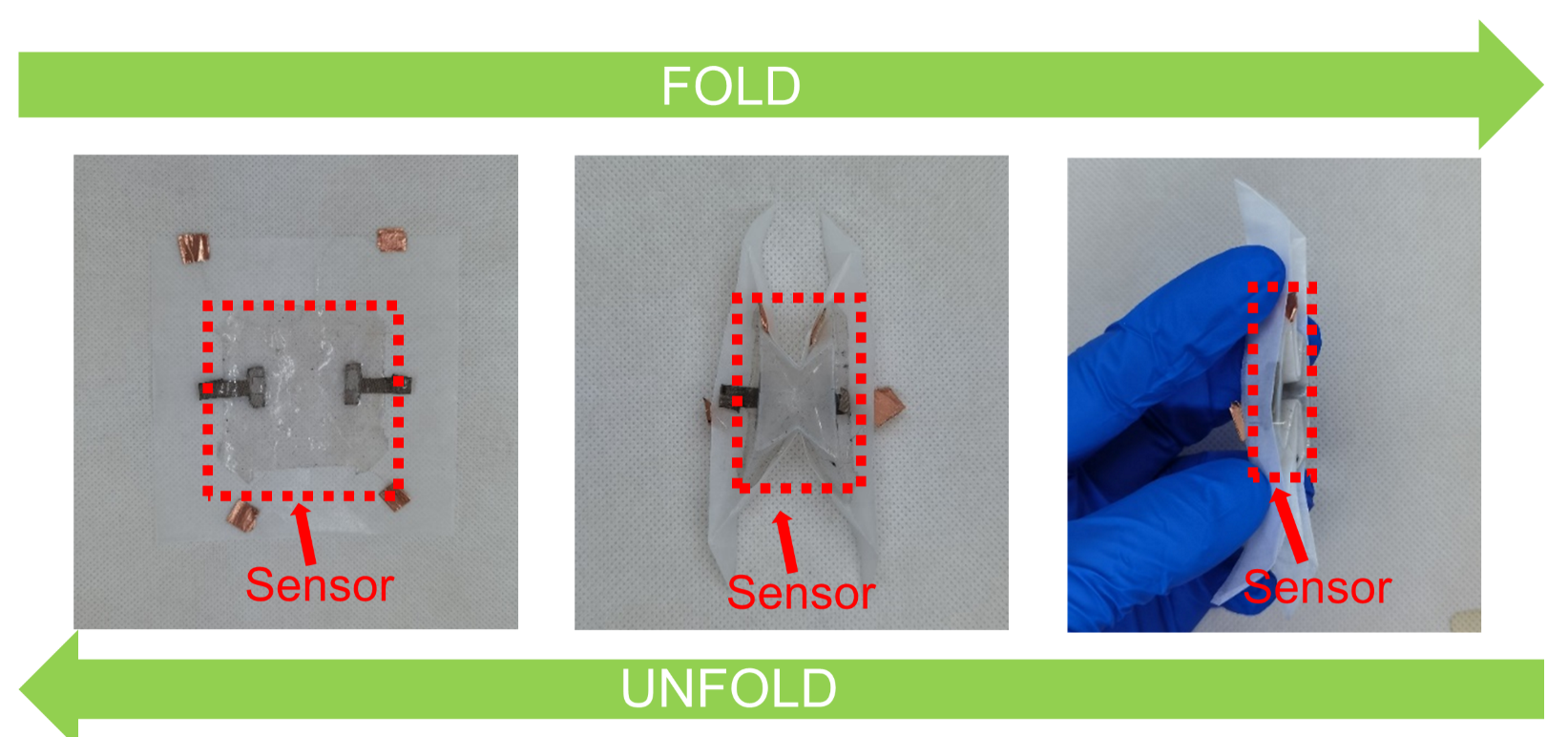


**Fig. S10** Photographs of multimodal e-skin being folded and unfolded


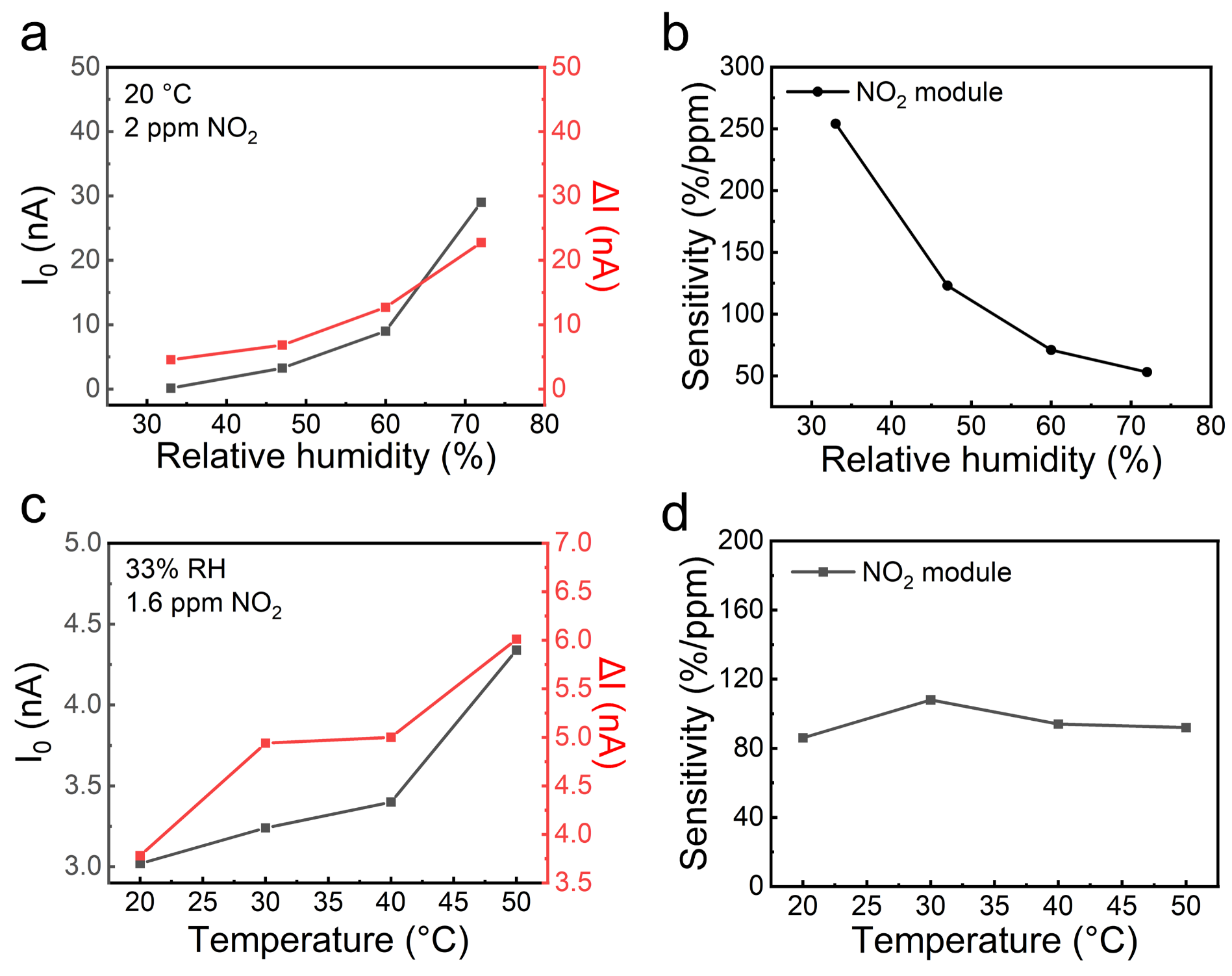


**Fig. S11** Changes of background current, response current and sensitivity of NO_2_ module with humidity or temperature. **a** Background current (I_0_) and response current (ΔI) to 2 ppm NO_2_ under different relative humidity. **b** Response sensitivity change curve of NO_2_ module under different humidity. **c** Background current and response current to 1.6 ppm NO_2_ at different temperatures. **d** Response sensitivity change curve of NO_2_ module at different temperatures


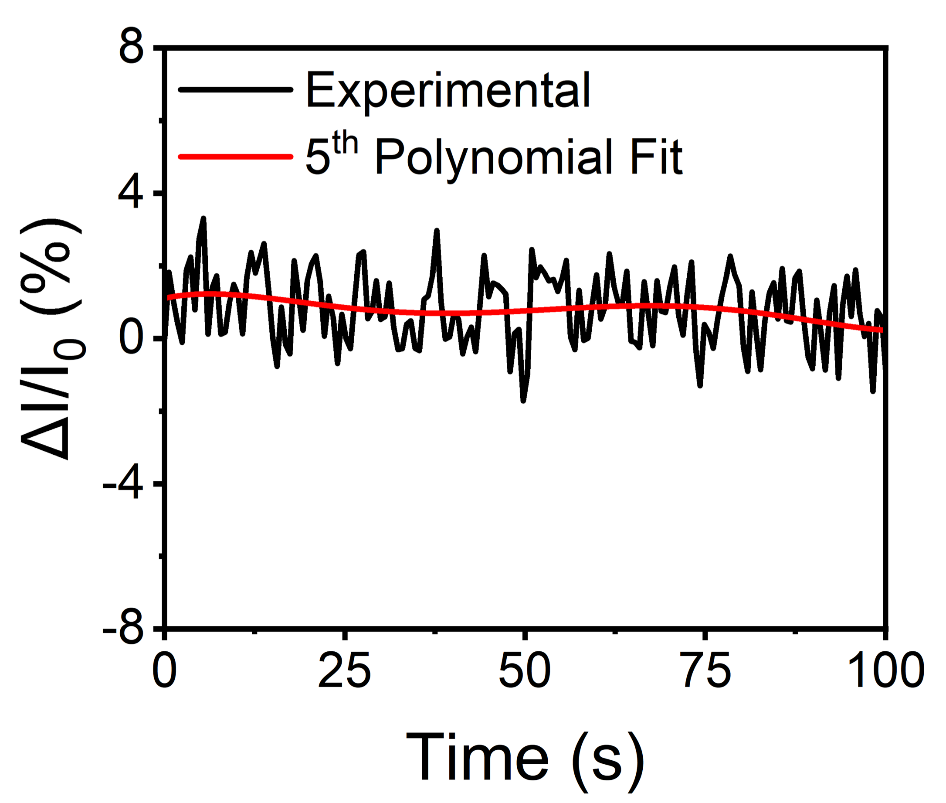


**Fig. S12** Background noise of the NO_2_ module and the 5^th^ order polynomial fitting curve of the noise before exposure to NO_2_


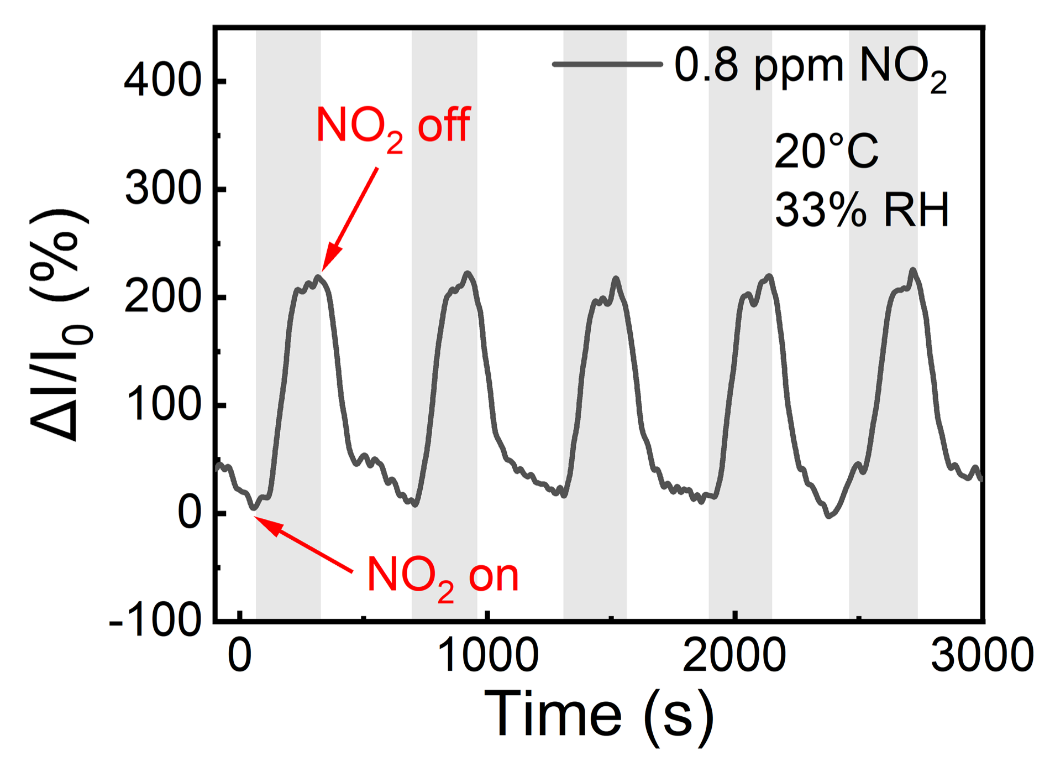


**Fig. S13** Dynamic response of the NO_2_ module to 0.8 ppm NO_2_ cycling

The process of data processing and heatmap drawing: The robot hand grabbed five kinds of objects, and each kind of object was grabbed for 20 times, obtaining 100 sets of samples with 15 features. The same class of samples is summed and averaged to represent the typical response value of each kind of object. Besides, the maximum value S_max_ and minimum value S_min_ of humidity response, temperature response and pressure/proximity response in 100 samples were extracted, respectively. The 15 features of each object class are normalized according to Equation 1, and then the normalized samples are drawn into heatmaps.

$\begin{aligned} S_{i,0-1}=\frac{S_{i}-S_{min}}{S_{max}-S_{min}}\#\left( S1 \right) \end{aligned}$
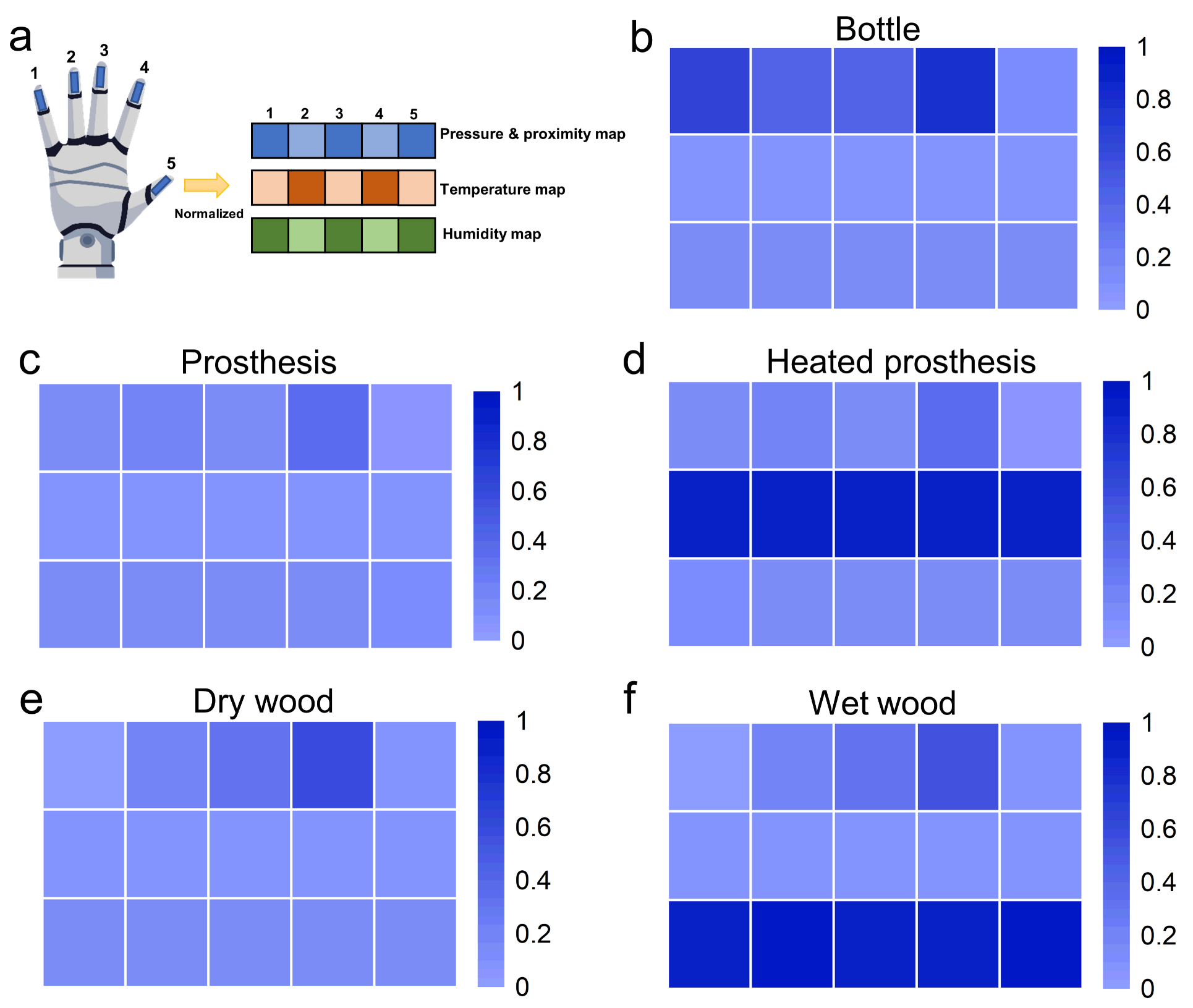


**Fig. S14** Visual images of the response signals recorded by the e-skins when the manipulator grasps different objects. **a** Schematic diagram of the robot hand with multimodal e-skin integrated on each finger and the corresponding normalized signal diagram of the e-skin. The robot hand grasps five types of objects, including **b** bottle, **c** prosthesis, **d** heated prosthesis, **e** dry wood, and **f** wet wood, with each object grasped for 20 times. The average value of the response signals collected when grasping each kind of object is shown in normalized heatmaps


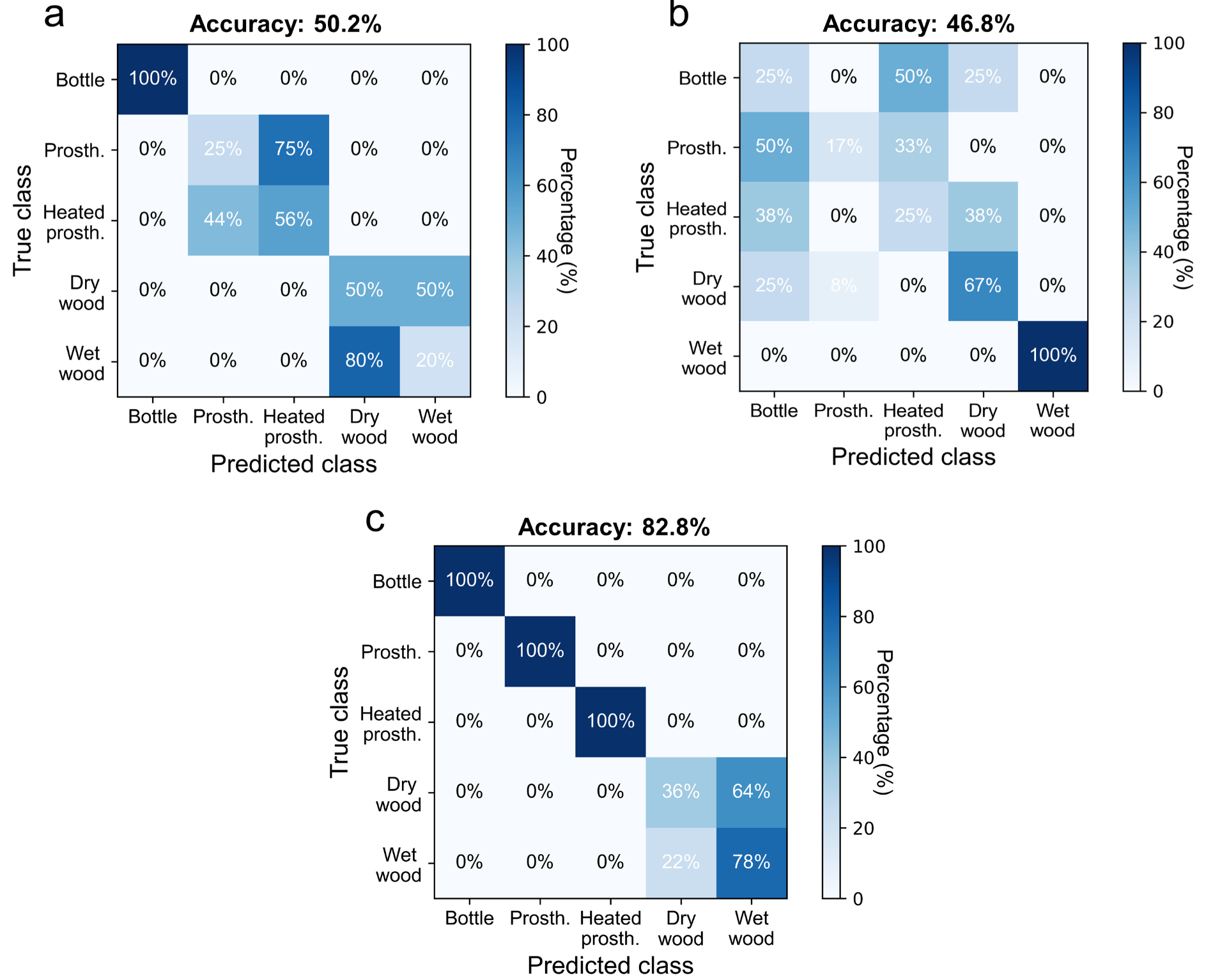


**Fig. S15** Classification test confusion matrix when different modules of the e-skin are blocked. **a** Classification test confusion matrix when using only pressure/proximity modules, **b** using only humidity modules and **c** using the combination of pressure/proximity and temperature modules of 5 multimodal e-skins


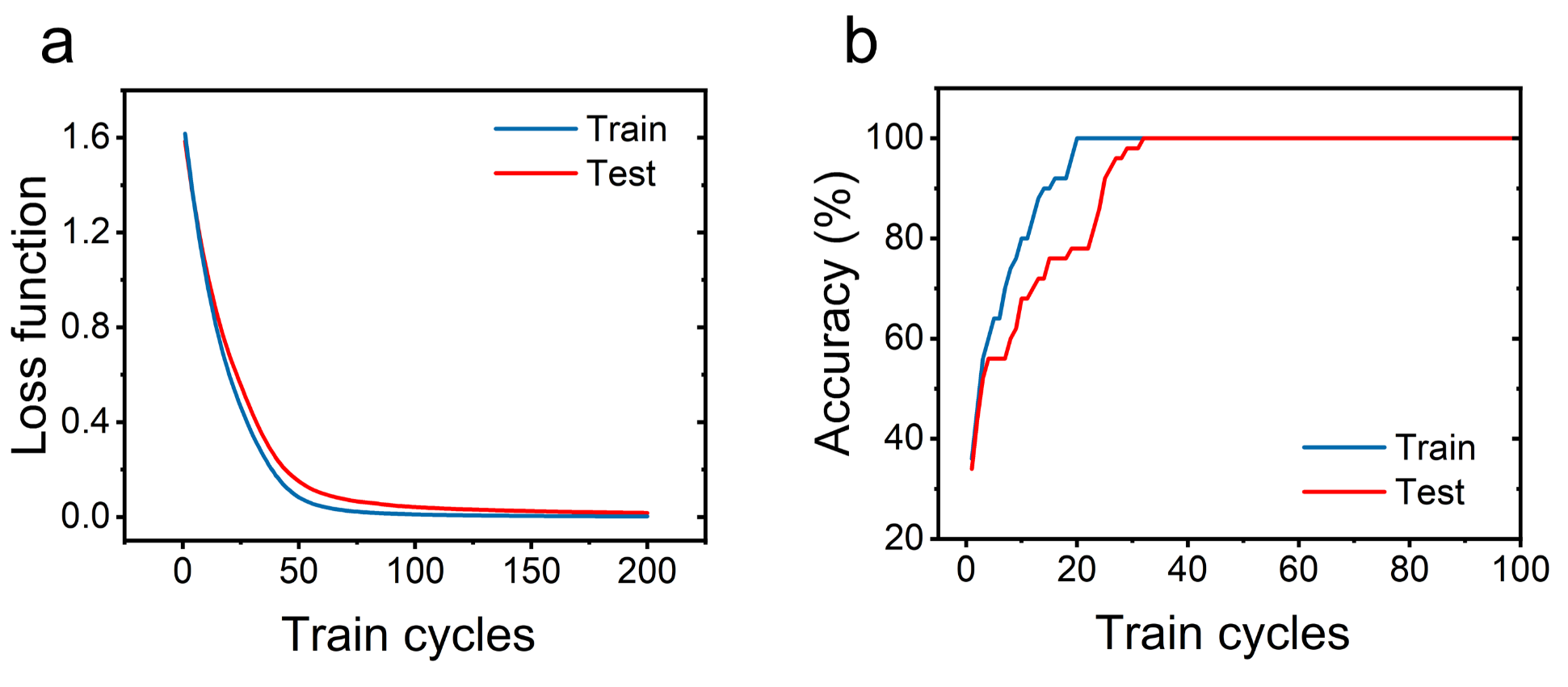


**Fig. S16** The degree of convergence of the model changes with the number of iterations. **a** The change curve of the loss function with the number of iterations during the DNN model training process. **b** The change curve of the object classification accuracy with the number of iterations during the DNN model training process

**Table S1** Performance comparison of hydrogel-based multimodal e-skins

| **Sensing material** | **Stimulus** | **Anti-freezing** | **Anti-drying** | **Stretchable** | **Transparent** | **Self-calibration** |
| --- | --- | --- | --- | --- | --- | --- |
| Silk fibroin/ poly(sulfobetaine)/Ca^2+^ hydrogel [S1] | Strain, proximity, temperature | Yes | Yes | Yes | Yes | No |
| PDMA-C18 hydrogel [S2] | Pressure, temperature | No | Yes | Yes | Yes | No |
| SAA hydrogel [S3] | Strain, temperature | No | Yes | Yes | Yes | No |
| PMP DN ICH hydrogel [S4] | Strain, temperature | Yes | Yes | Yes | No | No |
| PVA/Betaine-I^-^/I_3_^-^ [S5] | Strain, temperature | Yes | Yes | Yes | No | No |
| PVA-CNF  hydrogel[This work] | Pressure, temperature,  Humidity, NO_2_,  Proximity | Yes | Yes | Yes | Yes | Yes |

**Table S2** The relative humidity of the surrounding environment of different salt solutions at different temperatures

| Saturated salt solution | CH_3_COOK | MgCl_2_ | K_2_CO_3_ | NaBr | NaCl | K_2_SO_4_ |
| --- | --- | --- | --- | --- | --- | --- |
| 25 ℃ | 22.5% | 32.8% | 43.2% | 57.6% | 75.3% | 97.3% |
| 30 ℃ | 21.6% | 32.4% | 43.2% | 56% | 75.1% | 97% |
| 35 ℃ | 20.9% (#) | 32.1% | 43.6% (#) | 54.6% | 74.9% | 96.7% |
| 40 ℃ | 19.7% (*) | 31.6% | 43.4% (*) | 53.2% | 74.7% | 96.4% |
| 45 ℃ | 19.3% (#) | 31.1% | 43.2% (#) | 52% | 74.5% | 96.1% |

**Note:** Data marked by * is taken from Ref. [S6], data marked by # comes from Ref. [S7], and other data refer to Ref. [S8].

**Table S3** The relative humidity of dry and wet gas mixtures with different flow rate ratios

| **Dry gas** | **Wet gas** | **Relative humidity** |
| --- | --- | --- |
| 500 sccm | 0 sccm | 22.5% |
| 400 sccm | 100 sccm | 33% |
| 300 sccm | 200 sccm | 46.9% |
| 200 sccm | 300 sccm | 60.2% |
| 100 sccm | 400 sccm | 72.3% |
| 0 sccm | 500 sccm | 81.4% |

**Table S4** Comparison in the sensing targets, wireless sensing, stretchability, environmental object recognition, humidity response, pressure, and temperature sensitivity of various e-skins

| **Sensing materials** | **Stimulus** | **Wireless sensing** | **Stretchable** | **Environmental object recognition** | **Humidity**  **response** | **Pressure sensitivity (KPa^-1^)** | **Temp.**  **sensitivity (%/℃)** |
| --- | --- | --- | --- | --- | --- | --- | --- |
| Mxene/PVDF-TrFE [S9] | Pressure | No | Yes | No | / | 0.51 | / |
| PEDOT: PSS/  IGZO/  AgNF/AgNW [S10] | Pressure, temperature | No | Yes | No | / | 0.00178 (Maximum) | 0.03 |
| PU/PU@CNT [S11] | Pressure, temperature | No | No | No | / | 0.753 | 0.00284 |
| SWCNTs/TPU [S12] | Pressure, temperature | Yes | Yes | No | / | 0.02 | 1.65 |
| AAO/PEDOT: PSS/  CNT [S13] | Pressure | No | No | No | / | 0.67 | 1.41 |
| GO [S14] | Pressure, humidity, temperature | No | Yes | No | 27.5 | 0.002 | / |
| PVA/CA/AgNPs [S15] | Strain, humidity, temperature | No | Yes | No | ~0.9 | / | 0.076 |
| AgNWs/PDMS/  ZnS:Cu [S16] | Pressure | No | Yes | No | / | 1.28 (Maximum) | / |
| CB/Rgo [S17] | Strain, humidity, temperature, pressure | No | Yes | No | ~1.75 | 0.09 | 0.6 |
| PVA-CNF  Hydrogel [This work] | Humidity, pressure, proximity, temperature, NO_2_ | Yes | Yes | Yes | 238.2 | 0.57 | 9.38 |

The theoretical LOD of NO_2_ can be calculated by the following formula [S18]:

$$\begin{aligned} LOD=\frac{{3RMS}_{Noise}}{Slope}\#\left( S2 \right) \end{aligned}$$

Wherein RMS_noise_ is the root mean square derivation of the background current noise of the NO_2_ module in a pure nitrogen atmosphere, and the slope is the response sensitivity to NO_2_. According to the experimental results, the sensitivity of the NO_2_ module in an environment of 20 °C and 33% RH is 254%/ppm. The RMS_noise_ is calculated as 0.94% based on the background noise and the 5^th^ order polynomial fitting curve from Supplementary Figure 11. Finally, the theoretical LOD is calculated as low as 11.1 ppb.

**Table S5** The 5^th^ order polynomial fitting data used to calculate the LOD of the NO_2_ module. Y_i_ is the background response recorded by the NO_2_ module before exposure to NO_2_, and Y is the data obtained through 5th order polynomial fitting of the original curve

| **Time (s)** | **Y_i_-Y** | **(Y_i_-Y)^2^** |
| --- | --- | --- |
| 10 | 0.00144 | 2.074E-6 |
| 20 | 0.00765 | 5.852E-5 |
| 30 | -0.00213 | 4.537E-6 |
| 40 | -0.00036 | 1.296E-7 |
| 50 | -0.01723 | 2.969E-4 |
| 60 | -0.00248 | 6.150E-6 |
| 70 | 0.00666 | 4.436E-5 |
| 80 | -0.00915 | 8.372E-5 |
| 90 | -0.01296 | 1.680E-4 |
| 100 | 0.01146 | 1.313E-4 |

**Table S6** Performance comparison of representative NO_2_ sensors

| **Sensing material** | **LOD**  **(ppb)** | | **Operating condition** | **Sensitivity**  **(%/ppm)** | **Stretchable** | **Self-calibration** |
| --- | --- | --- | --- | --- | --- | --- |
| [EMIM]^+^[TFSI]^—^TPU [S19] | 250 | Room temperature | | 22.59%/5ppm | Yes | No |
| Ag/LIG [S20] | 4 | Room temperature | | 6.66‰/ppm | Yes | No |
| NiCo_2_O_4_/WO_3_ [S21] | 52 | 150 ℃ | | 379 | No | No |
| CuO thin films [S22] | 300 | 200 ℃ | | / | No | No |
| CuO/RGO [S23] | 50 | Room temperature | | 246.5 | No | No |
| WO_3_/S-rGO [S24] | 250 | Room temperature | | 5.548 | No | No |
| Pb_x_Cd_1−x_Se QD gel [S25] | 3 | Room temperature | | 60 | No | No |
| PVA-CNF  Hydrogel [This work] | 11.1 | Room temperature | | 254 | Yes | Yes |

**Supplementary Movies**

Movies supplementary material for this manuscript includes the following:

- **Movies S1:** Wireless detection of NO_x_ leakage events

**Supplementary References**

1. Z. Lei, W. Zhu, X. Zhang, X. Wang, P. Wu. Bio-inspired ionic skin for theranostics. Adv. Funct. Mater. **31**(8), 2008020 (2021). <https://doi.org/10.1002/adfm.202008020>
2. Z. Lei, Q. Wang, P. Wu. A multifunctional skin-like sensor based on a 3D printed thermo-responsive hydrogel. Mater. Horiz. **4**(4), 694-700 (2017). <https://doi.org/10.1039/C7MH00262A>
3. H. Huang, L. Han, X. Fu, Y. Wang, Z. Yang et al., Multiple stimuli responsive and identifiable zwitterionic ionic conductive hydrogel for bionic electronic skin. Adv. Electron. Mater. **6**(7), 2000239 (2020). <https://doi.org/10.1002/aelm.202000239>
4. W. Zhao, H. Zhou, W. Li, M. Chen, M. Zhou et al., An environment-tolerant ion-conducting double-network composite hydrogel for high-performance flexible electronic devices. Nano-Micro Lett. **16**(1), 99 (2024). <https://doi.org/10.1007/s40820-023-01311-2>
5. Z. Wang, N. Li, X. Yang, Z. Zhang, H. Zhang et al., Thermogalvanic hydrogel-based e-skin for self-powered on-body dual-modal temperature and strain sensing. Microsyst. Nanoeng. **10**(1), 55 (2024). <https://doi.org/10.1038/s41378-024-00693-6>
6. C. Arai, S. Hosaka, K. Murase, Y. Sano. Measurements of the relative humidity of saturated aqueous salt solutions. J. Chem. Eng. Jpn. **9**(4), 328-330 (1976). <https://doi.org/10.1252/JCEJ.9.328>
7. M. M. Alhussaini, H. A. Hassan, N. S. Ahmedzeki. Experimental investigation of moisture sorption isotherms for mefenamic acid tablets. Iraqi J. Chem. Pet. Eng. **21**(4), 11-20 (2020). <https://doi.org/10.31699/IJCPE.2020.4.2>
8. L. Greenspan. Humidity fixed points of binary saturated aqueous solutions. J. Res. Natl. Bur. Stand. A Phys. Chem. **81**(1), 89 (1977). <https://doi.org/10.6028/jres.081A.011>
9. S. Sharma, A. Chhetry, M. Sharifuzzaman, H. Yoon, J. Y. Park. Wearable capacitive pressure sensor based on MXene composite nanofibrous scaffolds for reliable human physiological signal acquisition. ACS Appl. Mater. Interfaces **12**(19), 22212-22224 (2020). <https://doi.org/10.1021/acsami.0c05819>
10. B. W. An, S. Heo, S. Ji, F. Bien, J.-U. Park. Transparent and flexible fingerprint sensor array with multiplexed detection of tactile pressure and skin temperature. Nat. Commun. **9**(1), 2458 (2018). <https://doi.org/10.1038/s41467-018-04906-1>
11. Z. Gao, Z. Lou, W. Han, G. Shen. A self-healable bifunctional electronic skin. ACS Appl. Mater. Interfaces **12**(21), 24339-24347 (2020). <https://doi.org/10.1021/acsami.0c05119>
12. Y. Zhang, Y. Zhao, W. Zhai, G. Zheng, Y. Ji et al., Multifunctional interlocked e-skin based on elastic micropattern array facilely prepared by hot-air-gun. Chem. Eng. J. **407**, 127960 (2021). <https://doi.org/10.1016/j.cej.2020.127960>
13. B. Liang, B. Huang, J. He, R. Yang, C. Zhao et al., Direct stamping multifunctional tactile sensor for pressure and temperature sensing. Nano Res. **4**(4), 1-7 (2022). <https://doi.org/10.1007/s12274-021-3906-x>
14. D. H. Ho, Q. Sun, S. Y. Kim, J. T. Han, D. H. Kim et al., Stretchable and multimodal all graphene electronic Skin. Adv. Mater. **28**(13), 2601-2608 (2016). <https://doi.org/10.1002/adma.201505739>
15. L. Chen, X. Chang, H. Wang, J. Chen, Y. Zhu. Stretchable and transparent multimodal electronic-skin sensors in detecting strain, temperature, and humidity. Nano Energy **96**, 107077 (2022). <https://doi.org/10.1016/j.nanoen.2022.107077>
16. Q. Tang, M. Zou, L. Chang, W. Guo. A super-flexible and transparent wood film/silver nanowire electrode for optical and capacitive dual-mode sensing wood-based electronic skin. Chem. Eng. J. **430**, 132152 (2022). <https://doi.org/10.1016/j.cej.2021.132152>
17. H. Liu, H. Xiang, Y. Wang, Z. Li, L. Qian et al., A flexible multimodal sensor that detects strain, humidity, temperature, and pressure with carbon black and reduced graphene oxide hierarchical composite on paper. ACS Appl. Mater. Interfaces **11**(43), 40613-40619 (2019). <https://doi.org/10.1021/acsami.9b13349>
18. A. Shrivastava, V. B. Gupta. Methods for the determination of limit of detection and limit of quantitation of the analytical methods. Chron. Young Sci. **2**(1), 21-25 (2011). <https://doi.org/10.4103/2229-5186.79345>
19. M. L. Jin, S. Park, H. Kweon, H.-J. Koh, M. Gao et al., Scalable superior chemical sensing performance of stretchable ionotronic skin via a π-hole receptor effect. Adv. Mater. **33**(13), 2007605 (2021). <https://doi.org/10.1002/adma.202007605>
20. L. Yang, G. Zheng, Y. Cao, C. Meng, Y. Li et al., Moisture-resistant, stretchable NO_x_ gas sensors based on laser-induced graphene for environmental monitoring and breath analysis. Microsyst. Nanoeng. **8**(1), 78 (2022). <https://doi.org/10.1038/s41378-022-00414-x>
21. Y. Hu, T. Li, J. Zhang, J. Guo, W. Wang et al., High-sensitive NO_2_ sensor based on p-NiCo_2_O_4_/n-WO_3_ heterojunctions. Sens. Actuators, B **352**, 130912 (2022). <https://doi.org/10.1016/j.snb.2021.130912>
22. A. Nanda, V. Singh, R. K. Jha, J. Sinha, S. Avasthi et al., Growth-temperature dependent unpassivated oxygen bonds determine the gas sensing abilities of chemical vapor deposition-grown CuO thin films. ACS Appl. Mater. Interfaces **13**(18), 21936-21943 (2021). <https://doi.org/10.1021/acsami.1c01085>
23. H. Bai, H. Guo, J. Wang, Y. Dong, B. Liu et al., A room-temperature NO_2_ gas sensor based on CuO nanoflakes modified with rGO nanosheets. Sens. Actuators, B **337**, 129783 (2021). <https://doi.org/10.1016/j.snb.2021.129783>
24. T. Wang, J. Hao, S. Zheng, Q. Sun, D. Zhang et al., Highly sensitive and rapidly responding room-temperature NO_2_ gas sensors based on WO_3_ nanorods/sulfonated graphene nanocomposites. Nano Res. **11**(2), 791-803 (2018). <https://doi.org/10.1007/s12274-017-1688-y>
25. X. Geng, S. Li, L. Mawella-Vithanage, T. Ma, M. Kilani et al., Atomically dispersed Pb ionic sites in PbCdSe quantum dot gels enhance room-temperature NO_2_ sensing. Nat. Commun. **12**(1), 4895 (2021). <https://doi.org/10.1038/s41467-021-25192-4>
